# SLAMF7 and SLAMF8 receptors shape human plasmacytoid dendritic cell responses to intracellular bacteria

**DOI:** 10.1172/JCI182467

**Published:** 2025-04-15

**Authors:** Joaquín Miguel Pellegrini, Anne Keriel, Laurent Gorvel, Sean Hanniffy, Vilma Arce-Gorvel, Mile Bosilkovski, Javier Solera, Stéphane Méresse, Sylvie Mémet, Jean-Pierre Gorvel

**Affiliations:** 1 Aix Marseille Université, CNRS, Inserm, Centre d’Immunologie de Marseille-Luminy (CIML), Marseille, France.; 2 Virulence Bactérienne et Infections Chroniques, U1047, Inserm, Université de Montpellier, Nîmes, France.; 3 Centre National de Reference des Brucella, Service de Microbiologie, Centre Hospitalier Universitaire de Nîmes, Nîmes, France.; 4 Tumor immunology laboratory, IBISA immunomonitoring platform, Cancer Research Center of Marseille, Marseille, France.; 5 University Clinic for Infectious Diseases and Febrile Conditions, Medical Faculty University “Ss Cyril and Methodius,” Skopje, North Macedonia.; 6 Internal Medicine Department, Albacete General Hospital, Albacete, Spain.; 7 University of Castilla–La Mancha at Albacete, Faculty of Medicine, Albacete, Spain.

**Keywords:** Immunology, Infectious disease, Microbiology, Bacterial infections, Dendritic cells

## Abstract

Plasmacytoid dendritic cells (pDCs), professional type I IFN–producing cells, have been implicated in host responses against bacterial infections. However, their role in host defense is debated, and the operating molecular mechanisms are unknown. Certain signaling lymphocyte activation molecule family (SLAMF) members act as microbial sensors and modulate immune functions in response to infection. Here, human blood transcriptomic analyses reveal the involvement of SLAMF7 and SLAMF8 in many infectious diseases, with elevated levels associated with type I IFN responses in salmonellosis and brucellosis patients. We further identify SLAMF7 and SLAMF8 as key regulators of human pDC function. They activate pDC maturation and cytokine production during infection with bacteria that induce acute (*Salmonella*) or chronic (*Brucella*) inflammation. SLAMF7 and SLAMF8 signal through NF-κB, IRF7, and STAT-1, and limit mitochondrial ROS accumulation upon *Salmonella* infection. Remarkably, this SLAMF7/8-dependent control of mitochondrial ROS levels favors bacterial persistence and NF-κB activation. Overall, our results unravel essential shared multifaceted roles of SLAMF7 and SLAMF8 in finely tuning human pDC responses to intracellular bacterial infections with potential for future diagnostic and therapeutic applications.

## Abstract 2

We identified the cell surface receptors SLAMF7 and SLAMF8 as key shared players in regulating human plasmacytoid cell response to infection and intracellular bacterial survival.

## Graphical abstract

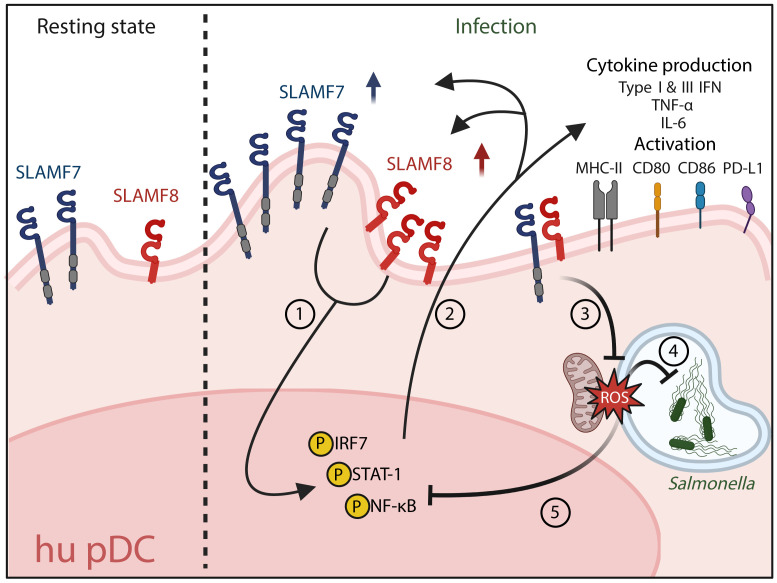

## Introduction

Intracellular bacteria pose significant challenges to the human immune system. Plasmacytoid dendritic cells (pDCs) are innate immune cells renowned for their role in antiviral responses, because of their exceptional capacity to produce large amounts of type I interferon (IFN) (1, 2). Their contribution to defense against bacteria is controversial. Specific deletion of pDCs during *Mycobacterium tuberculosis* infection reduces mycobacterial burden and infection pathogenesis in mice (3), in accordance with a detrimental role of pDCs during infection with *Citrobacter rodentium* (4), *Listeria monocytogenes* (5), or *Bordetella pertussis* (6). In contrast, pDC depletion worsens the outcome of *Legionella pneumophila* infection in mice (7) and delays post-acute *Klebsiella* pneumonia recovery (8). Human pDCs express receptors that regulate their activation and function (1). Yet the full spectrum of pDC receptors and mechanisms involved during bacterial infection remains obscure.

The signaling lymphocyte activation molecule family (SLAMF) of cell surface glycoproteins comprises 9 immunoreceptors widely expressed in myeloid and lymphoid cells (9), which have been implicated in diverse immune processes through homotypic interactions or heterotypic ones for SLAMF2 and SLAMF4. Some of these receptors serve as microbial sensors. As such, SLAMF1 controls the function of NK cells (10, 11), macrophages (12, 13), DCs (14), neutrophils (15), and hematopoietic stem cells (16) in response to infection; SLAMF2 and SLAMF6 also interact with bacterial constituents and regulate downstream inflammation (17–19). While some SLAMF members are expressed in pDCs, like SLAMF7 and SLAMF9 (20, 21), their role during infection is still to be unraveled.

Here, we reveal the participation of SLAMF7 (CS1, CRACC, CD319) and SLAMF8 (BLAME, CD353) in a broad spectrum of infectious diseases, showing increased levels in the blood of salmonellosis and brucellosis patients that correlate with a type I IFN response. We identify SLAMF7 and SLAMF8 as receptors that positively regulate human pDC function during infection with two types of intracellular bacteria known to drive either a strong acute inflammatory response (and high clearance except for 2%–5% of patients) (*Salmonella enterica* Typhimurium) (22) or a mild acute phase (undulant fever), often unnoticed, followed by a chronic multiorgan inflammatory stage of infection (*Brucella abortus*) (23). As such, to our knowledge, this study provides the first demonstration of an antibacterial response in pDCs orchestrated by the SLAMF7 and SLAM8 receptors through the activation of NF-κB, STAT-1, and IRF7 signaling and the regulation of mitochondrial reactive oxygen species production.

## Results

### Human blood transcriptomic signatures reveal changes in SLAMF7 and SLAMF8 expression levels in a large panel of infectious diseases.

**Figure 1:**
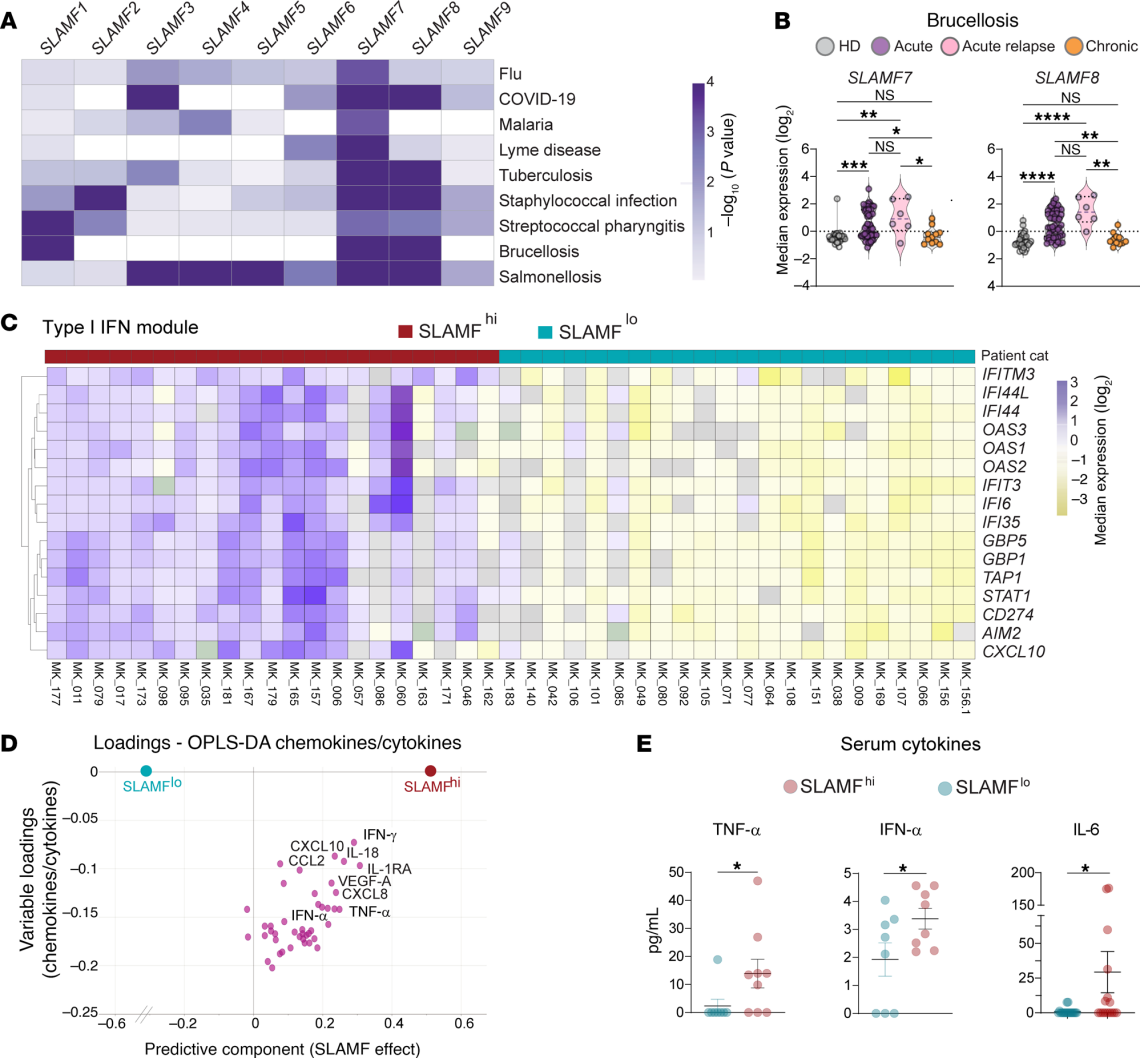
Blood transcriptomic signatures reveal the participation of SLAMF receptors during infection, and of SLAMF7 and SLAMF8 in brucellosis. (**A**) Heatmap from blood transcriptomics data showing the differential expression of SLAMF receptors between healthy donor (HD) individuals and patients suffering from flu (GSE100160), COVID-19 (GSE152075), malaria (GSE116149), Lyme disease (GSE145974), tuberculosis (GSE19491), staphylococcal infection (GSE100165), streptococcal pharyngitis (GSE158163), brucellosis (GSE69597), and salmonellosis (GSE113866). –Log_10_ *P* values are shown with intensity of the color representing the degree of variation from HDs. (**B** and **C**) RNA-Seq transcriptomic profiling obtained from whole-blood samples from HD controls or primary brucellosis patients in acute, acute relapse, or chronic phase of infection. (**B**) The median expression of *SLAMF7* and *SLAMF8* normalized counts is shown. *X* axis: HD controls, *n* = 36 (gray); brucellosis patients, acute, *n* = 54 (purple), acute with relapse, *n* = 6 (pink), chronic, *n* = 12 (orange). *Y* axis: log_2_ residual gene expression counts. Multiple-comparison Kruskal-Wallis test followed by post hoc Dunn’s test. *P < 0.05; **P < 0.01; ***P < 0.001; ****P < 0.0001. (**C**–**E**) “SLAMF” refers to SLAMF7 and SLAMF8. (**C**) Heatmap showing type I IFN module gene expression from whole-blood RNA-Seq data from SLAMF^hi^ (*n* = 21, dark red) and SLAMF^lo^ (*n* = 22, turquoise) brucellosis patients. (**D**) Orthogonal partial least squares discriminant analysis (OPLS-DA) loading plots of cytokines and chemokines measured in serum, harvested at first visit, from SLAMF^hi^ (*n* = 21, dark red) and SLAMF^lo^ (*n* = 22, turquoise) brucellosis patients. (**E**) Univariate analysis of selected cytokine concentration (pg/mL) in serum of SLAMF^hi^ (pink) and SLAMF^lo^ (cyan) brucellosis patients. TNF-α and IFN-α, SLAMF^hi^ (*n* = 9) and SLAMF^lo^ (*n* = 8); IL-6, SLAMF^hi^ (*n* = 16) and SLAMF^lo^ (*n* = 18). Significant differences are shown (Mann-Whitney test), *P < 0.05.

We first conducted a comprehensive analysis of available datasets of blood transcriptomics from healthy individuals and patients suffering from a wide range of infections, including viral (flu, COVID-19), parasitic (malaria), and bacterial (Lyme disease, tuberculosis, staphylococcal infection, streptococcal pharyngitis, brucellosis, salmonellosis) diseases, and highlighted the putative contribution of each of the 9 SLAMF receptor family members, given the known ability of some of them to recognize and respond to pathogens. We identified in these unrelated infections, and especially in bacterial diseases (except Lyme borreliosis for *SLAMF8*), significant differences between healthy donors (HDs) and infected individuals in the RNA expression of *SLAMF7* and *SLAMF8* (Figure 1A and Supplemental Figure 1A; supplemental material available online with this article; https://doi.org/10.1172/JCI182467DS1).

### The SLAM-associated signature in brucellosis patients correlates with type I IFN response.

We next assessed the participation of SLAMF7 and SLAMF8 in human host defense against *Brucella*, an intracellular bacterium whose peculiarity is to evade the host immune response and establish a chronic infection (24). We analyzed genome-wide transcriptomes that we generated by bulk blood RNA-Seq from HDs and brucellosis patients at different stages of the disease (in acute, acute with relapse, or chronic stages at their first visit) (25, 26). Several genes of the SLAM family (*SLAMF1*, *SLAMF7*, and *SLAMF8*) were overexpressed in acute but not in chronic brucellosis patients, unlike stable *TLR9* gene expression (Figure 1B and Supplemental Figure 1B). Both *SLAMF7* and *SLAMF8* RNA levels were positively correlated in HDs and acute brucellosis patients (Supplemental Figure 1C), in spite of the different functions ascribed thus far to SLAMF7 and SLAMF8 (27–32). The discriminative value of SLAMF was investigated by receiver operating characteristic curve analysis (Supplemental Figure 1D), giving a receiver operating area under the curve (ROAUC) of 0.7650 (95% CI, 0.6380–0.8919; *P* = 0.0074) for *SLAMF7* and of 0.8736 (95% CI, 0.7764–0.9708; *P* = 0.0002) for *SLAMF8* transcript levels, and confirming the correlation with brucellosis. Categorization of brucellosis patients according to their SLAMF7 and SLAMF8 receptor expression (Supplemental Figure 1E) indicated disparity in the global blood transcriptome of these groups, with *MAPK11* and *VAMP5* as top genes differentially enriched in SLAMF^hi^ patients, and *SESN3* and *MYBPH* in the SLAMF^lo^ group (Supplemental Figure 1F), despite any differences in dominant symptoms and comorbidities (Supplemental Figure 1G). Importantly, these data unveiled a type I IFN signature in SLAMF^hi^ patients as compared with SLAMF^lo^ individuals (Figure 1C). Cytokine and chemokine levels potentially associated with the clinical outcome of *Brucella* infection were measured in the sera of SLAMF^hi^ and SLAMF^lo^ brucellosis patients and analyzed by orthogonal partial least squares regression. Cytokine secretion was enriched in sera from SLAMF^hi^ patients, in which top discriminatory analytes included IFN-γ, IL-18, CXCL10, and IL-1RA (Figure 1D). Significantly higher levels of TNF-α, IFN-α, and IL-6 also distinguished SLAMF^hi^ from SLAMF^lo^ brucellosis patients (Figure 1E). Altogether these data identify SLAMF7 and SLAMF8 as hallmarks of acute brucellosis and link their overexpression to type I IFN responses.

### The SLAM-associated signature in salmonellosis patients correlates with type I IFN response.

**Figure 2:**
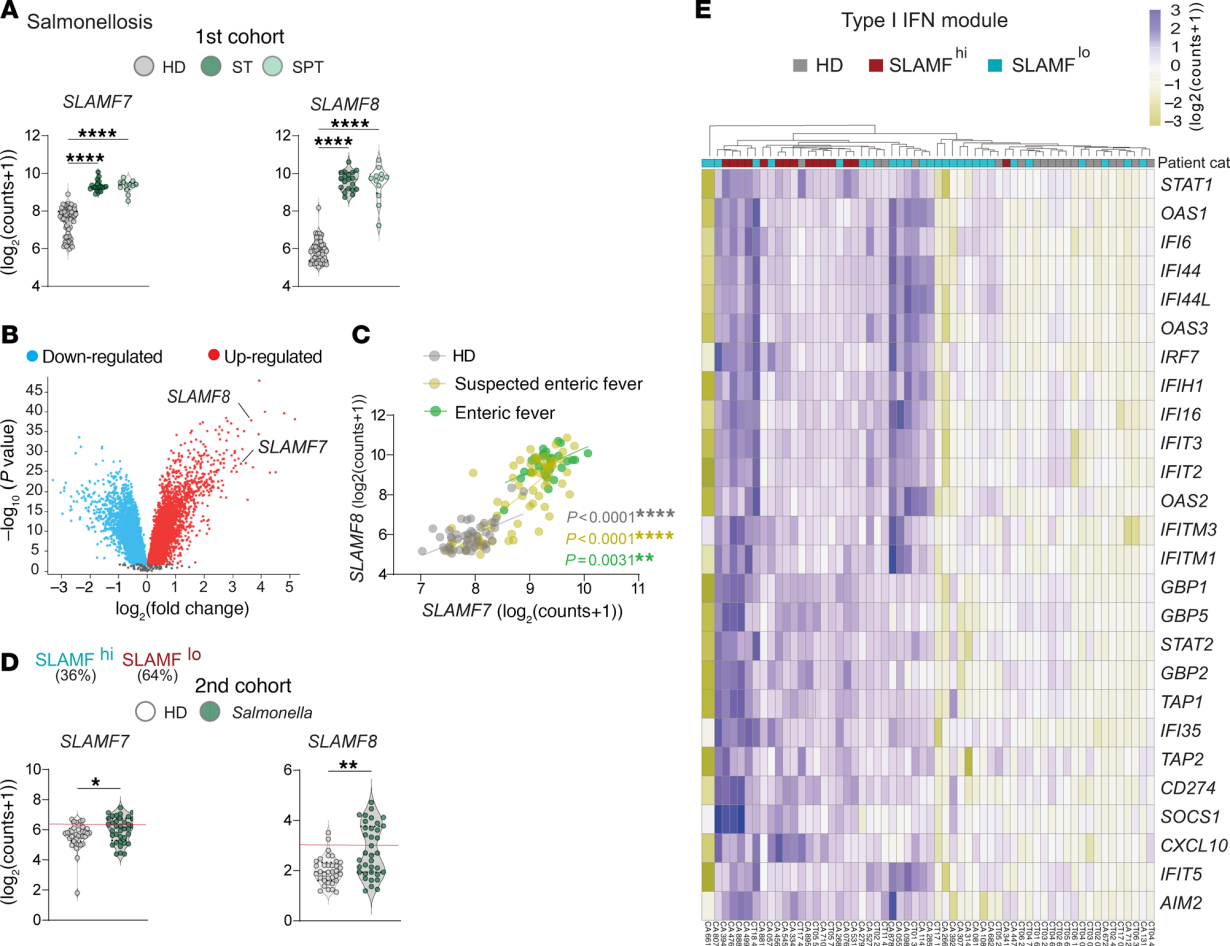
Blood transcriptomic signatures reveal the participation of SLAMF7 and SLAMF8 in salmonellosis. (**A**) *SLAMF7* and *SLAMF8* normalized counts obtained by transcriptomic profiling analysis from whole-blood samples from a first cohort (GSE113866) composed of HD (*n* = 68, gray) controls and enteric fever patients with positive cultures for *Salmonella enterica* Typhi (ST; *n* = 19, dark green) or *Salmonella enterica* Paratyphi (SPT; *n* = 12, light green). Significant differences are shown (multiple-comparison Kruskal-Wallis test followed by post hoc Dunn’s test). ****P < 0.0001. (**B**) Volcano plot showing differentially expressed genes between HDs and salmonellosis patients from the first cohort (upregulated, red; and downregulated, cyan). (**C**) Correlation between *SLAMF7* and *SLAMF8* RNA counts across all groups of individuals, i.e., HDs (grays, *n* = 47) and patients with suspected enteric fever (*n* = 71, yellow) or culture-confirmed enteric fever (*n* = 30, green). Non-parametric Spearman’s correlation test. (**D** and **E**) “SLAMF” refers to SLAMF7 and SLAMF8. (**D**) *SLAMF7* and *SLAMF8* normalized counts obtained by transcriptomic profiling analysis from whole-blood samples from a second cohort (GSE69529) composed of HD (*n* = 35, gray) controls and age-matched patients with *Salmonella* gastroenteritis (*n* = 36, green). Multiple-comparison Kruskal-Wallis test followed by post hoc Dunn’s test. *P < 0.05; **P < 0.01. (**E**) Heatmap showing type I IFN module gene expression from whole-blood RNA-Seq data analysis of the second cohort with HDs (*n* = 35, gray) and patients with *Salmonella* gastroenteritis categorized according to their *SLAMF7* and *SLAMF8* expression in SLAMF^hi^ (*n* = 15, dark red) and SLAMF^lo^ (*n* = 26, turquoise) individuals.

Since *SLAMF7* and *SLAMF8* genes were differentially expressed in the blood transcriptome of patients suffering from infection with *Salmonella*, a paradigm of bacteria that drive acute inflammation (Figure 1A), we analyzed in depth a first cohort of salmonellosis patients comprising enteric fever patients with positive cultures for both *Salmonella enterica* Typhi and *Salmonella enterica* Paratyphi (33). Significantly higher *SLAMF7* and *SLAMF8* RNA counts were found in whole blood of infected individuals as compared with HDs (Figure 2A), which ranked them in the volcano plot among the 50 most differentially expressed genes (Figure 2B). A significant positive correlation between *SLAMF7* and *SLAMF8* RNA counts appeared across all groups of individuals, i.e., HDs, culture-confirmed enteric fever patients, and even suspected enteric fever patients (Figure 2C). A second cohort of patients, comprising children under 10 years of age afflicted with diarrheal diseases (34), confirmed a significant upregulation of *SLAMF7* and *SLAMF8* in those diagnosed with *Salmonella* gastroenteritis (Figure 2D). Applying recursive partitioning analysis to define the optimal *SLAMF7* and *SLAMF8* expression cutoffs allowed us to categorize these salmonellosis patients into SLAMF^hi^ (*n* = 15, 36%) and SLAMF^lo^ (*n* = 26, 64%) subgroups, with similar demographic and clinical features (Supplemental Figure 1H). Strikingly in both cohorts, *Salmonella*-infected patients showed an elevated type I IFN signature as compared with HDs, which was particularly enriched in SLAMF^hi^ patients (Figure 2E and Supplemental Figure 1, I and J).

### SLAMF7 and SLAMF8 are overexpressed in human pDCs upon TLR7/8 stimulation.

**Figure 3:**
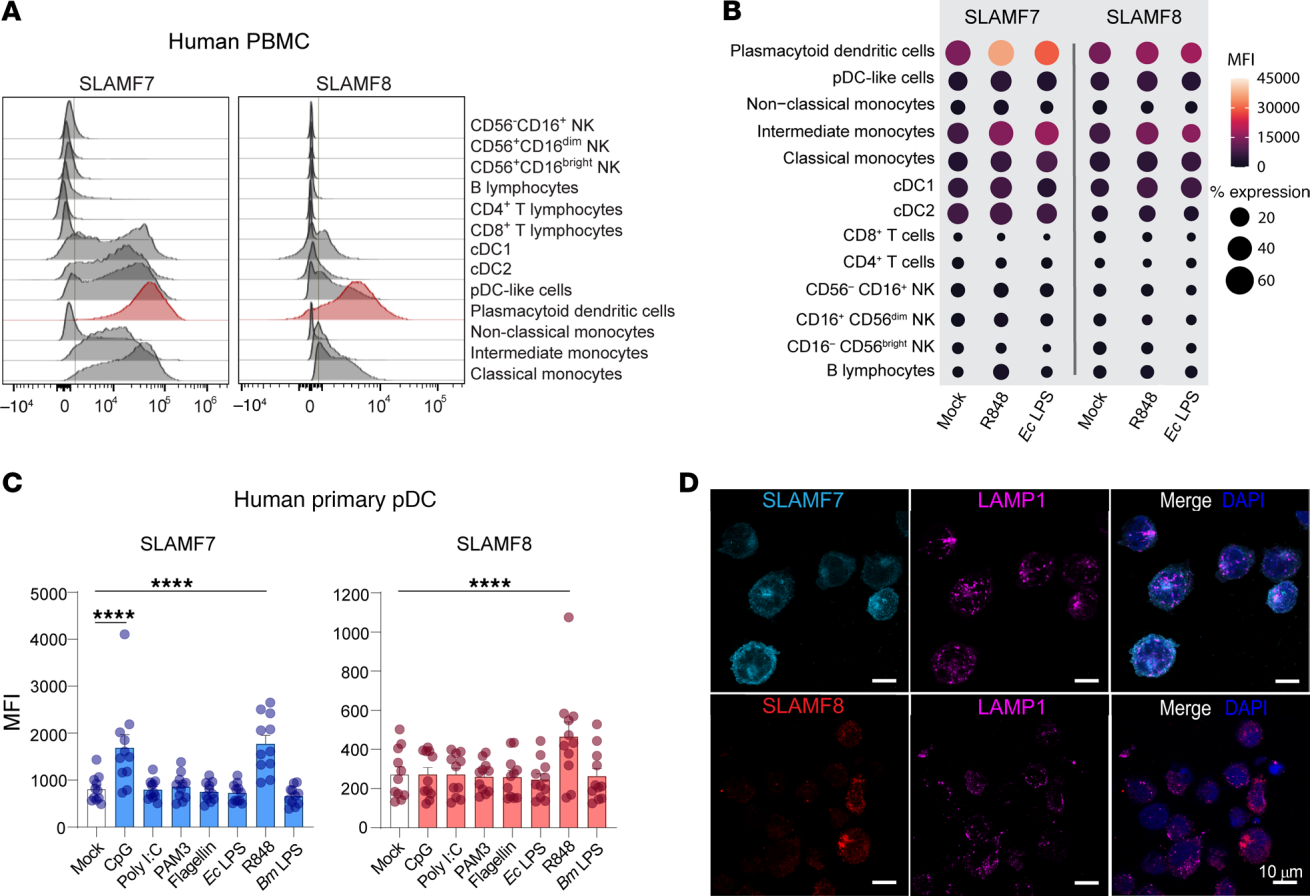
SLAMF7 and SLAMF8 are expressed at high levels in pDCs. (**A** and **B**) SLAMF7 and SLAMF8 surface expression on the different cell types of PBMCs from HDs was analyzed by spectral flow cytometry. (**A**) Representative histograms at the resting state with positivity indicated by a vertical gray bar are shown. *n* = 12. (**B**) Analysis 24 hours after treatment (or not) of PBMCs with R848 or *E*. *coli* LPS (100 ng/mL) for 24 hours. One donor per *n*; *n* = 12. Dot plots represent the median fluorescence intensity (MFI; color) and the percentage of expression (size) in a determined cell type. Color and size codes are indicated on the right. (**C**) Purified human primary pDCs were stimulated with CpG (TLR9 ligand, 100 ng/mL), poly(I:C) (TLR3 ligand, 100 ng/mL), PAM3CSK4 (TLR1/2 ligand, 100 ng/mL), flagellin (TLR5 ligand, 100 ng/mL), *E*. *coli* LPS (TLR4 ligand, 100 ng/mL), R848 (TLR7/8 ligand, 100 ng/mL), or *B*. *melitensis* LPS (10 μg/mL) for 24 hours. Then, SLAMF7 and SLAMF8 levels of expression were evaluated by flow cytometry. Histograms presenting MFI of SLAMF7 and SLAMF8 are shown. Mean ± SD. One donor per *n*; *n* = 11. Significant differences are indicated. For each individual experiment, values for CpG or R848 conditions were always above those of the mock-treated cells for SLAMF7, and above (*n* = 10) or similar (*n* = 1) for SLAMF8 MFI. One-way ANOVA followed by Dunnett’s multiple-comparison test. ****P < 0.0001. (**D**) Representative confocal microscopy images of CAL-1 cells in basal conditions stained for SLAMF7 (turquoise, top left and right panels), SLAMF8 (red, bottom left and right panels), LAMP-1 (magenta, middle and right panels), and nuclei (DAPI, blue, right panels). Scale bars: 10 μm. *n* = 5.

Considering that SLAMF receptors are predominantly expressed in innate and adaptive immune cells, we then assessed SLAMF7 and SLAMF8 expression in the discrete immune cell populations of peripheral blood mononuclear cells (PBMCs) from HDs at steady state and after stimulation with TLR ligands by spectral flow cytometry. pDCs were the cell subpopulation expressing the highest basal levels of SLAMF7 or SLAMF8, with an elevated expression close to that of monocytes after stimulation with the ligand of TLR7/8, R848, or the ligand of TLR4, the prototypical *Escherichia coli* LPS (Figure 3, A and B). Notably, SLAMF7 and SLAMF8 were also constitutively expressed but at much lower levels in pDC-like cells and conditional DC 1 (cDC1) and conventional DC 2 (cDC2) DCs with a limited effect of TLR7/8 or TLR4 stimulation (Figure 3, A and B).

The association of a type I IFN signature with SLAMF7 and SLAMF8 upregulation in brucellosis and salmonellosis patients together with their high expression in pDCs and the known role of these cells in type I IFN production led us to explore the expression of these 2 surface receptors in purified human pDCs at resting state and after exposure to various stimuli comprising TLR ligands and bacterial components (Figure 3C and Supplemental Figure 2A). Sixty-six percent and 10% of primary pDCs expressed basal levels of SLAMF7 and SLAMF8, respectively (Supplemental Figure 2A). TLR7 stimulation with R848 significantly increased the expression levels of both receptors in human primary pDCs, unlike other TLR ligands or bacterial components such as poly(I:C), PAM3, flagellin, or *E*. *coli* or *Brucella melitensis* LPS (Figure 3C and Supplemental Figure 2A). The TLR9 ligand CpG upregulated the surface expression of SLAMF7 only (Figure 3C). In CAL-1 cells, a human pDC cell line that recapitulates many features of its primary counterparts, SLAMF7 and SLAMF8 were constitutively expressed in 50% and 5% of cells, respectively (Supplemental Figure 2B). Confocal microscopy revealed an intracellular pool of both molecules, located in vesicles negative for the late compartment marker LAMP1 (Figure 3D). As in primary pDCs, CpG or R848 triggered a significant rise in the percentage of SLAMF7^+^ CAL-1 cells, while only R848 increased the SLAMF8^+^ CAL-1 cell percentage (Supplemental Figure 2B). Neither other TLR ligands nor exposure to outer membrane vesicles (OMVs) derived from *B*. *abortus* wild-type (WT) or lacking the outer membrane protein Omp25 (Supplemental Figure 2B), which we previously demonstrated to interact with SLAMF1 in murine DCs (14), modified the proportion of SLAMF7^+^ and SLAMF8^+^ cells in this human pDC cell line. Altogether, we show that the SLAMF7 and SLAMF8 receptors are expressed in human pDCs and upregulated by a TLR7/8 agonist.

### Human pDC infection by Brucella or Salmonella increases SLAMF7 and SLAMF8 expression.

**Figure 4:**
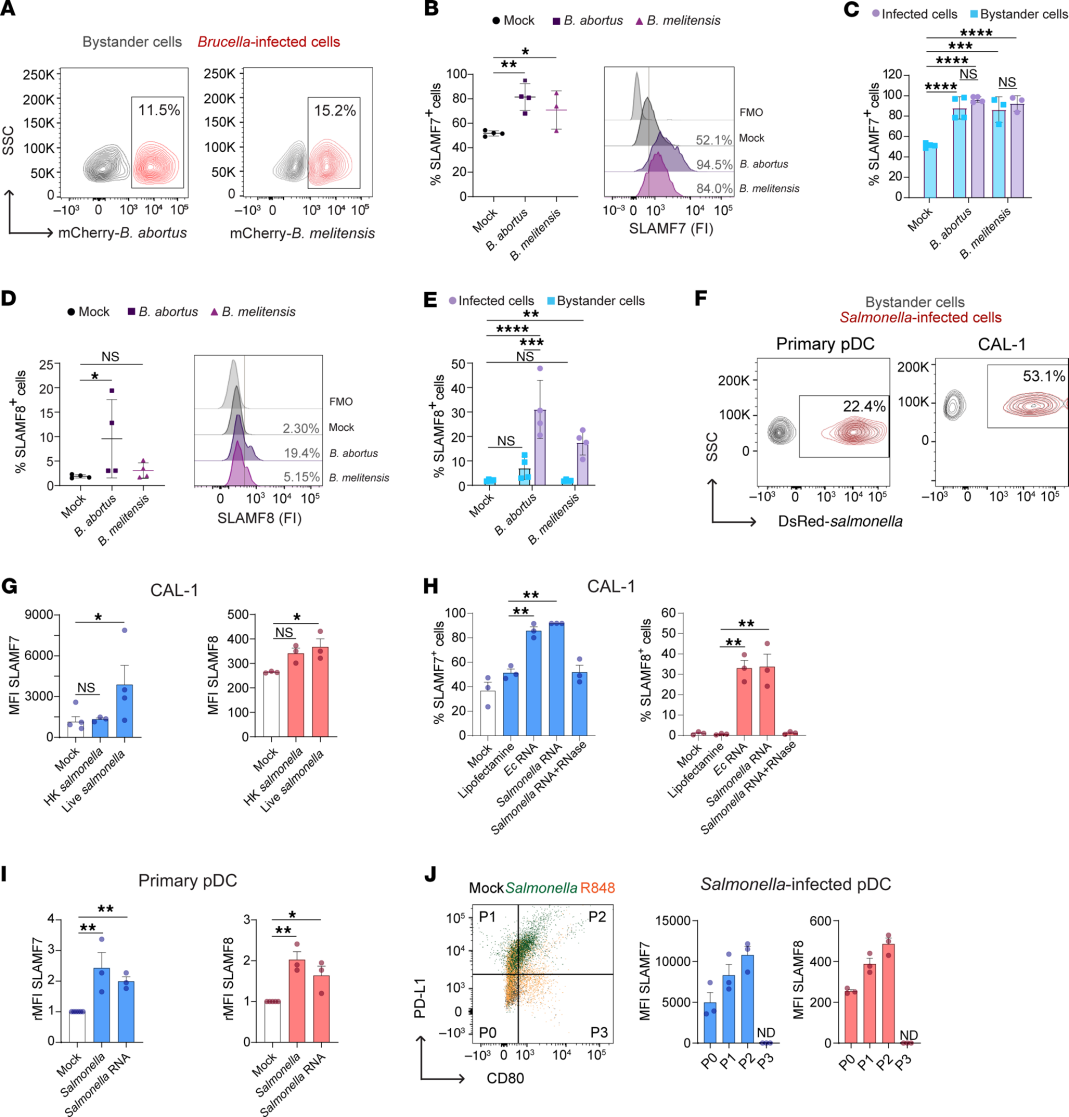
Human pDC infection by *Brucella* or *Salmonella* induces SLAMF7 and SLAMF8 expression. (**A**) Flow cytometry contour plot graphs showing percentage of CAL-1 cells infected (red) with mCherry–*Brucella abortus* or *melitensis* at 48 hours post-infection (p.i.) (MOI 5,000). *n* = 4. (**B**–**E**) SLAMF7 or SLAMF8 expression in CAL-1 cells at 48 hours p.i. with *B*. *abortus* (purple) or *melitensis* (pink-purple) (MOI 5,000). (**B**) Left: Percentage of total SLAMF7^+^ cells. Each dot represents an independent experiment. Mean ± SD. *n* = 3–4. Right: Histograms of SLAMF7 fluorescence intensity (FI). Vertical positivity bar (gray). (**C**) In cells from **B**, percentages of SLAMF7^+^ infected (light purple) or bystander (light blue) cells. (**D**) As in **B**, but for SLAMF8. (**E**) As in **C**, but for SLAMF8. (**F**) Flow cytometry contour plot graphs showing percentage of primary pDCs and CAL-1 cells infected (red) with DsRed–*Salmonella* *enterica* Typhimurium at 24 hours p.i. (MOI 25). *n* = 12. (**G**) SLAMF7 and SLAMF8 MFI of heat-killed (HK) or live *Salmonella*–infected or mock CAL-1 cells at 24 hours p.i. Mean ± SD. *n* = 3–4. (**H**) Percentage of SLAMF7^+^ and SLAMF8^+^ CAL-1 cells at 24 hours after stimulation with RNA from *E*. *coli* (*Ec*) or *Salmonella* with or without RNase. Mean ± SD. *n* = 3. (**I**) Relative MFI of SLAMF7 and SLAMF8 to mock-treated cells, put arbitrarily at 1, of human primary pDCs at 24 hours after *Salmonella* RNA stimulation or infection. Mean ± SD. *n* = 3. (**J**) Left: Dot plot graph showing CD80 and PD-L1 expression of mock-treated (black), R848-stimulated (orange), or *Salmonella*-infected (green) primary pDCs defining 4 subsets, P0–P3. Right: SLAMF7 (left) and SLAMF8 (right) MFI in pDC subsets at 24 hours p.i. with *Salmonella*. Mean ± SD. *n* = 3. Statistical differences were all calculated using 1-way ANOVA followed by Dunnett’s multiple-comparison test. *P < 0.05; **P < 0.01; ***P < 0.001; ****P < 0.0001.

To determine whether SLAMF7 and SLAMF8 expression may vary in human pDCs during brucellosis, we infected CAL-1 cells with *B*. *abortus* or *B*. *melitensis*, the 2 main pathogenic species causing infection in humans. Both mCherry-*Brucella* species efficiently infected CAL-1 cells (Figure 4A and Supplemental Figure 2C). mCherry–*B*. *abortus* or –*B*. *melitensis* infection highly increased SLAMF7 surface expression (Figure 4B) at 48 hours post-infection (p.i.) in infected as well as bystander cells (Figure 4C), suggesting that soluble factor(s) mediate SLAMF7 overexpression in non-infected cells. In contrast, only the percentage of SLAMF8^+^ infected cells (and not bystander cells) was significantly augmented by *B*. *abortus* or *B*. *melitensis* infection, thereby explaining the slight rise in the total SLAMF8^+^ CAL-1 cell percentage at 48 hours p.i. (Figure 4, D and E). Altogether, human pDCs upregulate via different mechanisms the levels of SLAMF7 and SLAMF8 upon *Brucella* infection.

We next evaluated SLAMF7 and SLAMF8 expression in pDCs upon *Salmonella enterica* Typhimurium infection. By using a DsRed-expressing bacterium, we showed by flow cytometry and confocal microscopy that *Salmonella* efficiently infected human primary pDCs as well as CAL-1 cells (Figure 4F and Supplemental Figure 2D). Surface expression of SLAMF7 and SLAMF8, but not SLAMF1, significantly increased in CAL-1 cells infected with live *Salmonella*, as well as the percentage of SLAMF7^+^ and SLAMF8^+^ infected cells (Figure 4G and Supplemental Figure 2, E and F). The fact that stimulation with heat-killed *Salmonella* had no effect (Figure 4G) opened up a possible involvement of the ligand of TLR7, the viability-associated pathogen-associated molecular pattern (*vita*-PAMP) microbial RNA (35). Indeed, prokaryotic RNA purified from *E*. *coli* or *S*. Typhimurium increased the percentage of SLAMF7^+^ and SLAMF8^+^ CAL-1 cells in an RNase-dependent manner (Figure 4H), as well as the surface levels of SLAMF7 and SLAMF8, in primary human pDCs, like in the case of *Salmonella* infection (Figure 4I). This confirms the role of RNA sensing in SLAMF7 and SLAMF8 upregulation in human pDCs. Notably, both receptors were enriched in the P1 (PD-L1^+^CD80^–^) and P2 (PD-L1^+^CD80^+^) pDC subsets (Figure 4J), specialized in type I IFN production for the former and displaying both innate and adaptive functions for the latter (36). Altogether, we establish that human pDCs upregulate the levels of both SLAMF7 and SLAMF8 upon infection with live *Salmonella*, in a process requiring bacterial viability and prokaryotic RNA recognition.

### SLAMF7 and SLAMF8 are required for full activation of Brucella-infected pDCs.

To explore SLAMF7 and SLAMF8 function in human pDCs, we generated specific knockdown (KD) CAL-1 cells by lentiviral transduction of short hairpin RNA (shRNA) targeting these receptors (Supplemental Figure 3A). Stable clones were selected based on GFP expression and specific SLAMF receptor silencing as measured by flow cytometry and quantitative reverse transcriptase PCR (Supplemental Figure 3, B–D). The basal level of SLAMF7 was downregulated in SLAMF8-KD cells compared with that in non-targeting shRNA control (shCTRL) or CAL-1 cells (Supplemental Figure 3, C and D). After R848 stimulation, SLAMF8 expression was also significantly reduced in SLAMF7-KD CAL-1 cells; conversely, silencing SLAMF8 decreased SLAMF7 surface expression (Supplemental Figure 3D). These observations were confirmed at the protein and mRNA levels upon infection (Supplemental Figure 3C). Functionally, knockdown of SLAMF7 or SLAMF8 in CAL-1 cells significantly abrogated the increased expression of maturation markers (HLA-DR, CD80, CD86) and of the coinhibitory molecule PD-L1 24 hours after treatment with R848 (Supplemental Figure 3E).

**Figure 5:**
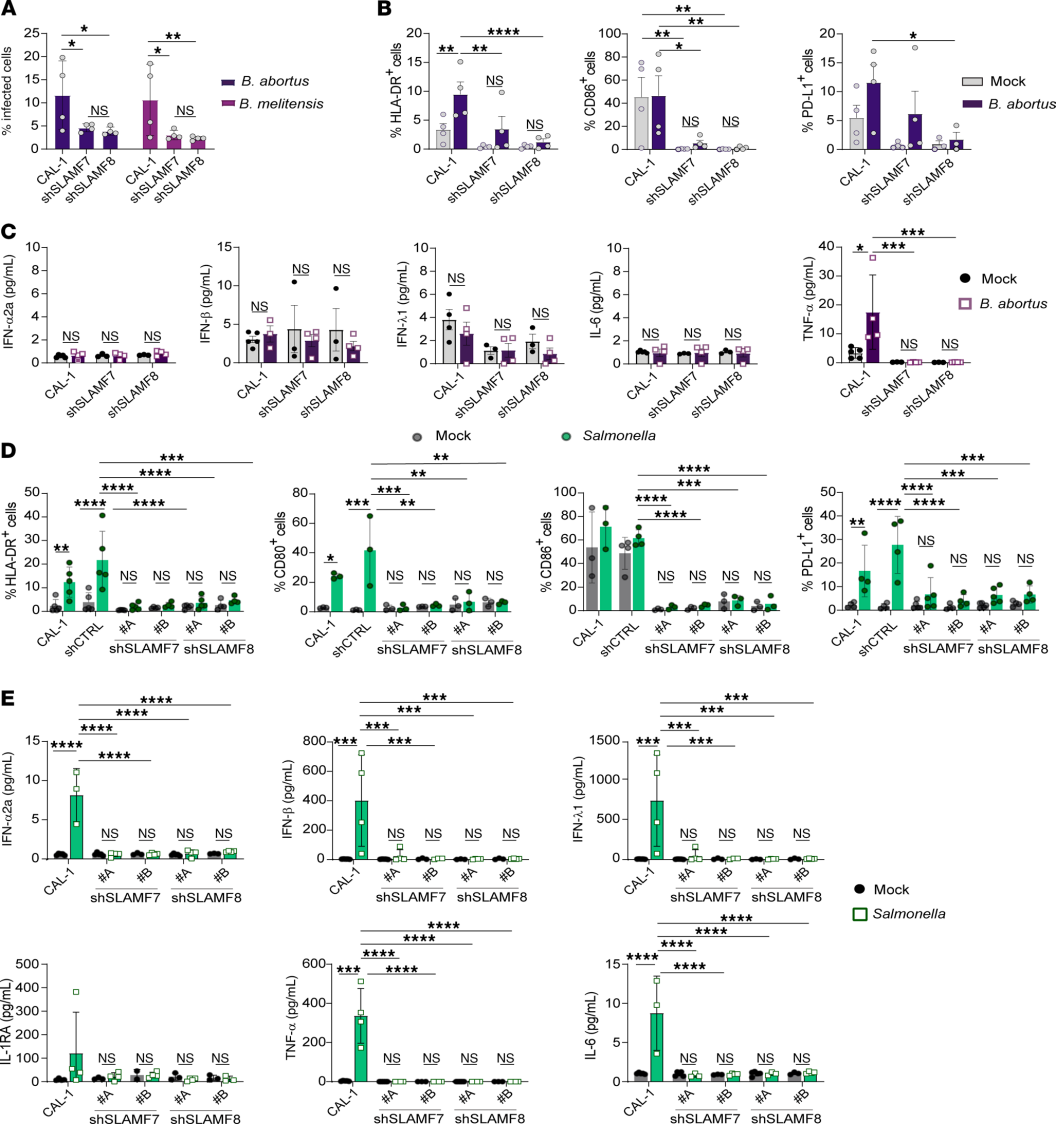
SLAMF7 and SLAMF8 regulate pDC activation and cytokine secretion during intracellular bacterial infection. (**A**) CAL-1 and SLAMF-silenced CAL-1 cells were infected with mCherry–*B*. *abortus* (dark purple) or –*B*. *melitensis* (purple) (MOI 5,000), and cells harboring bacteria were analyzed at 48 hours p.i. by flow cytometry. Column graphs showing mCherry-*Brucella*^+^ cells. Mean ± SD. *n* = 4. (**B** and **C**) CAL-1 and SLAMF-KD cells were infected with mCherry–*B*. *abortus* (dark purple) (MOI 5,000) for 48 hours. (**B**) Expression of human pDC activation markers was determined by flow cytometry. Column graphs showing percentages of HLA-DR^+^ (left), CD80^+^ (middle), and PD-L1^+^ (right) cells. Mean ± SD. *n* = 4. (**C**) Cytokine secretion was determined in culture supernatants using multiplex assay. Column graphs showing cytokine concentration (pg/mL). Each individual point represents 1 independent experiment. Mean ± SD. *n* = 4. (**D** and **E**) CAL-1 cells and CAL-1 cells stably transduced with SLAMF (2 independent clones, A and B, analyzed per type of SLAM-KD cells, SLAMF7 or SLAMF8, as well as 1 control clone stably transduced with a short hairpin empty vector, shCTRL) were infected with *Salmonella* Typhimurium (MOI 25, green) or not (black) for 24 hours. (**D**) Expression of activation markers was determined by flow cytometry. Column graphs showing percentages of HLA-DR^+^ (left), CD80^+^ (left center), CD86^+^ (right center), and PD-L1^+^ (right) cells. Each individual point represents 1 independent experiment. Mean ± SD. *n* = 3–4 except 5 for HLA-DR. (**E**) Cytokine secretion was determined in culture supernatants using multiplex assay. Column graphs showing cytokine concentration (pg/mL). Each individual point represents 1 independent experiment. Mean ± SD. *n* = 3–4. Statistical differences were all calculated using 2-way ANOVA followed by Šidák’s multiple-comparison test or non-parametric Mann-Whitney test for unpaired samples. *P < 0.05; **P < 0.01; ***P < 0.001; ****P < 0.0001.

Then, we asked about the possible role of the pDC SLAMF7 and SLAMF8 receptors in *Brucella* virulence. Silencing SLAMF7 or SLAMF8 significantly reduced the percentage of *B*. *abortus*– and *B*. *melitensis*–infected CAL-1 cells 48 hours p.i. (Figure 5A). CAL-1 cells after *B*. *abortus* infection harbored, as expected given *Brucella*’s extended immunosuppressive abilities, a moderate level of pDC activation, with a low increase of maturation marker expression and secretion of TNF-α, the only detectable cytokine by multiplex assay (Figure 5, B and C, and Supplemental Figure 3, F and G). Silencing of SLAMF7 or SLAMF8 significantly diminished the proportion of HLA-DR– and CD86-expressing pDCs, as well as of cells expressing PD-L1, but only in SLAMF8-silenced pDCs for the latter (Figure 5B). In addition, *B*. *abortus*–elicited TNF-α secretion was completely abolished in the SLAMF7- or SLAMF8-KD cells at 48 hours p.i. (Figure 5C). Altogether, these results indicate that SLAMF7 and SLAMF8 are required for full activation of human pDCs during *Brucella* infection.

### SLAMF7 and SLAMF8 are essential for activation and cytokine secretion of Salmonella-infected human pDCs.

**Figure 6:**
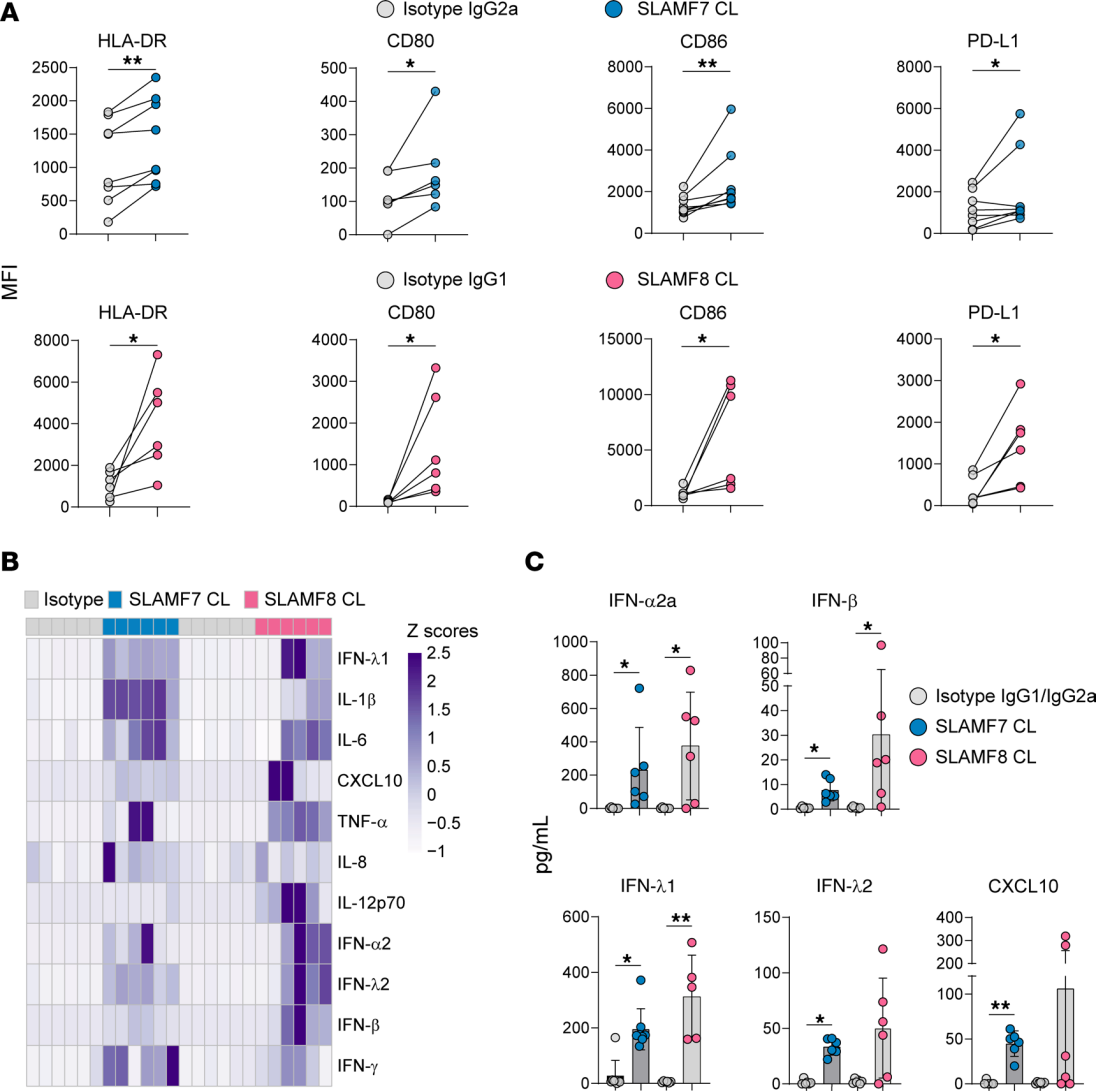
SLAMF7 or SLAMF8 engagement in human primary pDCs elicits strong cell activation, as well as high cytokine and type I and III IFN secretion. (**A**–**C**) Purified human primary pDCs from healthy individuals were infected with *Salmonella* Typhimurium (MOI of 25) and, after 1 hour, washes, and addition of gentamicin, SLAMF7 or SLAMF8 were engaged by cross-linking with 10 μg/mL of a specific antibody (anti-SLAMF7, blue filled circles, or anti-SLAMF8, pink filled circles) or its isotype control (empty circles) for 1 hour. Cells were then analyzed at 24 hours p.i. (**A**) Expression (MFI) of human pDC activation markers (HLA-DR, left; CD80, middle left; CD86, middle right; and PD-L1, right) was determined by flow cytometry. Each individual point represents 1 independent experiment. Mean ± SD. One donor per *n*; *n* = 6. Significant differences are indicated. Paired 2-tailed *t* test. *P < 0.05; **P < 0.01. (**B**) Heatmap of selected cytokines and chemokines, analyzed by multiplex flow cytometry in cell culture supernatants. *Z* scores of cytokine/chemokine concentrations are shown with intensity of the color representing the degree of variation in each condition from HD pDCs treated with the isotype control antibodies. One donor per *n*; *n* = 6. (**C**) Secretion of different IFN type I and III subspecies and of CXCL10 (pg/mL) determined in cell culture supernatants using multiplex flow cytometry. Each individual point represents 1 independent experiment. Mean ± SD. One donor per *n*; *n* = 6. Significant differences are shown. Paired 2-tailed *t* test. *P < 0.05; **P < 0.01. No *P* value indicates not significant.

To determine whether these two SLAMF receptors affect human pDC function during *Salmonella* infection, we analyzed our KD and shCTRL clones and CAL-1 cells at 24 hours p.i. Silencing of SLAMF7 and SLAMF8 hindered the maturation of human pDCs infected with *Salmonella* compared with that of shCTRL or CAL-1 cells (Figure 5D), without affecting cell death (Supplemental Figure 3H). Consistently, cytokine secretion by CAL-1 cells, measured by multiplex assay, of type I and III IFN (IFN-α2, IFN-β, IFN-λ1), IL-1RA, TNF-α, and IL-6 was completely abolished in SLAMF7- or SLAMF8-KD cells (Figure 5E). No IFN-γ, IL-1β, nor IL-10 production was detected at this time point whatever the cell type (Supplemental Figure 3I). The impaired activation in SLAMF7- or SLAMF8-KD cells was also evidenced at the RNA level, with significantly reduced expression levels of inflammatory cytokine genes like *IFNB* and *TNFA*, of an IFN-stimulated gene (*ISG15*), and of the gene encoding IκBα, *NFKBIA* (Supplemental Figure 3J). Conversely, the mRNA levels of the antiinflammatory cytokine IL-10 were greatly elevated (>500-fold) upon silencing of SLAMF7 or SLAMF8, in comparison with CAL-1 or shCTRL cells (Supplemental Figure 3J). To analyze the role of SLAMF7 and SLAMF8 in human primary pDCs, we next examined the effect of the direct engagement of one of these two receptors by a specific antibody or its isotype control on the response of these cells to *Salmonella* infection (Figure 6). Cross-linking of SLAMF7 or SLAMF8 in human primary pDCs significantly increased the expression of maturation markers (HLA-DR, CD80, CD86) and of the coinhibitory molecule PD-L1 at 24 hours after infection with *S*. Typhimurium when compared with the levels observed in the isotype control antibody condition (Figure 6A). It also resulted in the over-secretion of cytokines (Figure 6B), and in particular of type I IFN (IFN-α, IFN-β, IFN-λ1, IFN-λ2) and CXCL10, in the culture medium (Figure 6, B and C). Collectively, these findings indicate that SLAMF7 and SLAMF8 play a positive regulatory role in human pDC responses to bacterial infection by promoting cell activation and proinflammatory cytokine and IFN type I secretion.

### SLAMF7 and SLAMF8 engagement causes Salmonella persistence in human pDCs.

**Figure 7:**
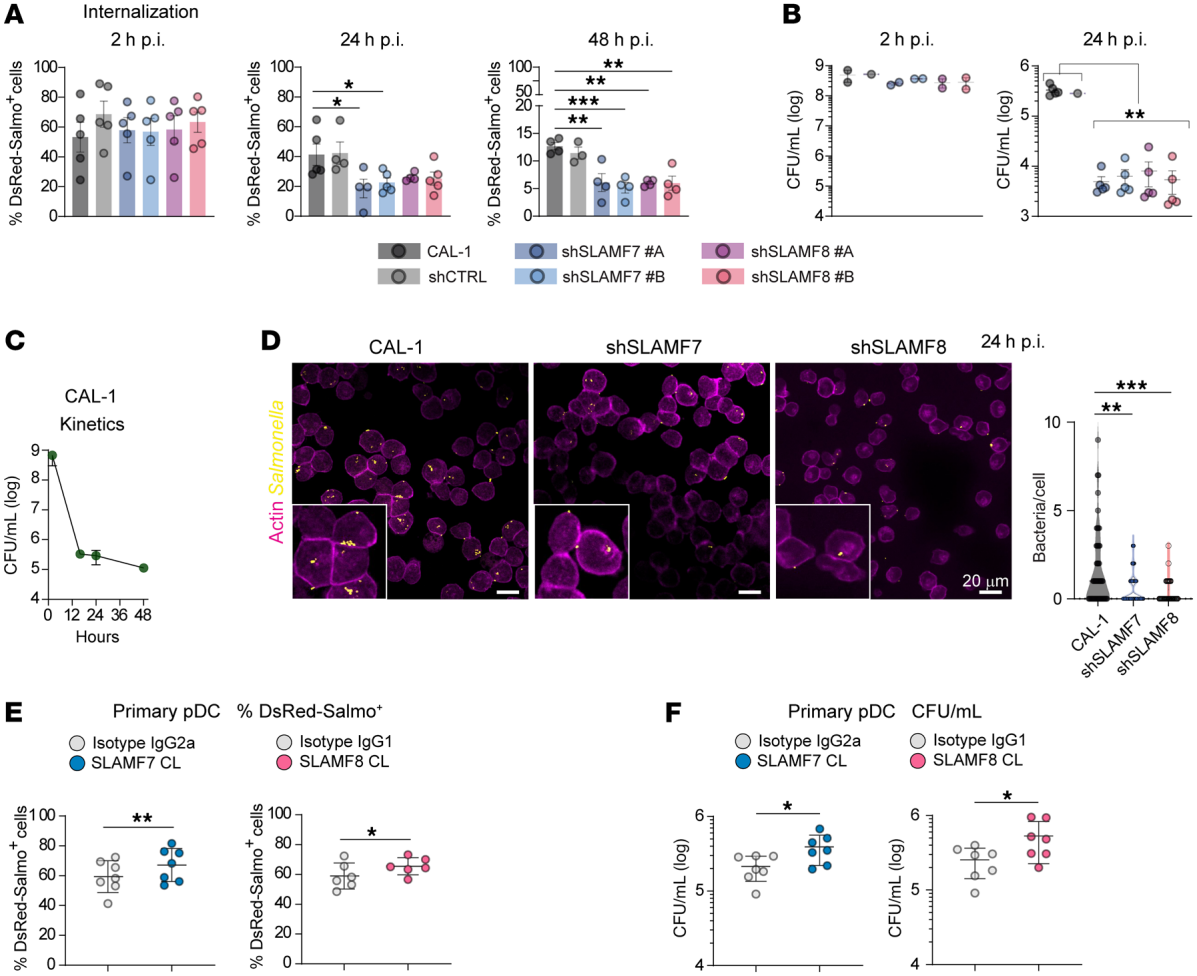
SLAMF7 and SLAMF8 limit *Salmonella* elimination by human pDCs. (**A**–**C**) CAL-1 and SLAMF7- or SLAMF8-silenced (2 clones, A and B, per SLAMF-KD type) and shCTRL CAL-1 cells were infected with DsRed–*S*. Typhimurium (MOI 25) for the indicated times p.i. One-way ANOVA followed by Dunnett’s multiple-comparison test. (**A**) Percentages of DsRed-*Salmonella*^+^ cells determined by flow cytometry at 2 hours (Internalization), 24 hours, and 48 hours p.i. Each individual point represents 1 independent experiment. Mean ± SD. *n* = 4–5. (**B**) Bacterial intracellular burden (CFU/mL, log) at indicated time points. Each individual point represents 1 independent experiment. Mean ± SD. *n* = 2 (2 hours), *n* = 5 (24 hours). (**C**) Kinetics experiment. *Salmonella* loads (CFU/mL, log) at indicated time points. Each individual point represents 1 independent experiment. Mean ± SD. *n* = 4. (**D**) Left: Confocal images of CAL-1 and SLAMF-silenced cells infected with DsRed–*S*. Typhimurium at 24 hours p.i. *Salmonella*, yellow; actin, purple. Scale bars: 20 μm. *n* = 3. Right: Violin plots depicting the number of bacteria per cell. Pooled data (total cells counted, 50–66). Multiple-comparison Kruskal-Wallis test followed by post hoc Dunn’s test. (**E**) Primary human cells were infected with DsRed–*S*. Typhimurium (MOI 25) and, after 1 hour, washes, and addition of gentamicin, SLAMF7 or SLAMF8 were engaged by cross-linking with a specific antibody or its isotype control. Percentages of DsRed-*Salmonella*^+^ cells at 24 hours p.i. Each individual point represents 1 independent experiment. Mean ± SD. One donor per *n*; SLAMF7, *n* = 7; SLAMF8, *n* = 6. Paired 2-tailed *t* test. (**F**) *Salmonella* loads (CFU/mL, log) at 24 hours p.i. in human primary pDCs treated as in **E**. Each individual point represents 1 independent experiment. Mean ± SD. One donor per *n*; *n* = 7. Paired 2-tailed *t* test. For all panels, significant differences are shown. *P < 0.05; **P < 0.01; ***P < 0.001. No *P* value indicates not significant.

Phagocytosis and elimination of hematopoietic tumor cells by macrophages require SLAMF7 independently of SLAM-associated adaptors (37). Hence, we asked whether SLAMF7 or SLAMF8 receptors contribute to bacteria phagocytosis by human pDCs. Evaluation of bacteria internalization at 2 hours p.i., in contrast to later time points, disclosed no differences in the percentage of DsRed-*Salmonella*^+^ cells between CAL-1 and SLAMF-KD cells (Figure 7A), and in CFU numbers (Figure 7B), excluding a role for SLAMF7 or SLAMF8 in bacteria phagocytosis by pDCs. Kinetics experiments showed that intracellular bacterial loads decreased over time in CAL-1 cells, suggesting that pDCs actively kill internalized *Salmonella* (Figure 7C). However, SLAMF7- or SLAMF8-KD cells eliminated bacteria more efficiently than CAL-1 and shCTRL cells, as evidenced by a reduced percentage of DsRed-*Salmonella*^+^ cells and lower CFU and number of bacteria per cell at 24 hours p.i. (Figure 7, A, B, and D). This decrease of DsRed-*Salmonella*^+^ cells when SLAMF7 or SLAMF8 was silenced in CAL-1 cells reversed to an increase of DsRed-*Salmonella*^+^ cells when one of these two receptors was engaged by cross-linking with a specific antibody in primary human pDCs (Figure 7E). Higher CFUs were also observed in this latter condition (Figure 7F). Altogether, these findings in CAL-1 cells and primary pDCs demonstrate that SLAMF7 or SLAMF8 engagement fosters *Salmonella* persistence in human pDCs.

### SLAMF7 and SLAMF8 facilitate Salmonella persistence in human pDCs by limiting mitochondrial ROS accumulation.

**Figure 8:**
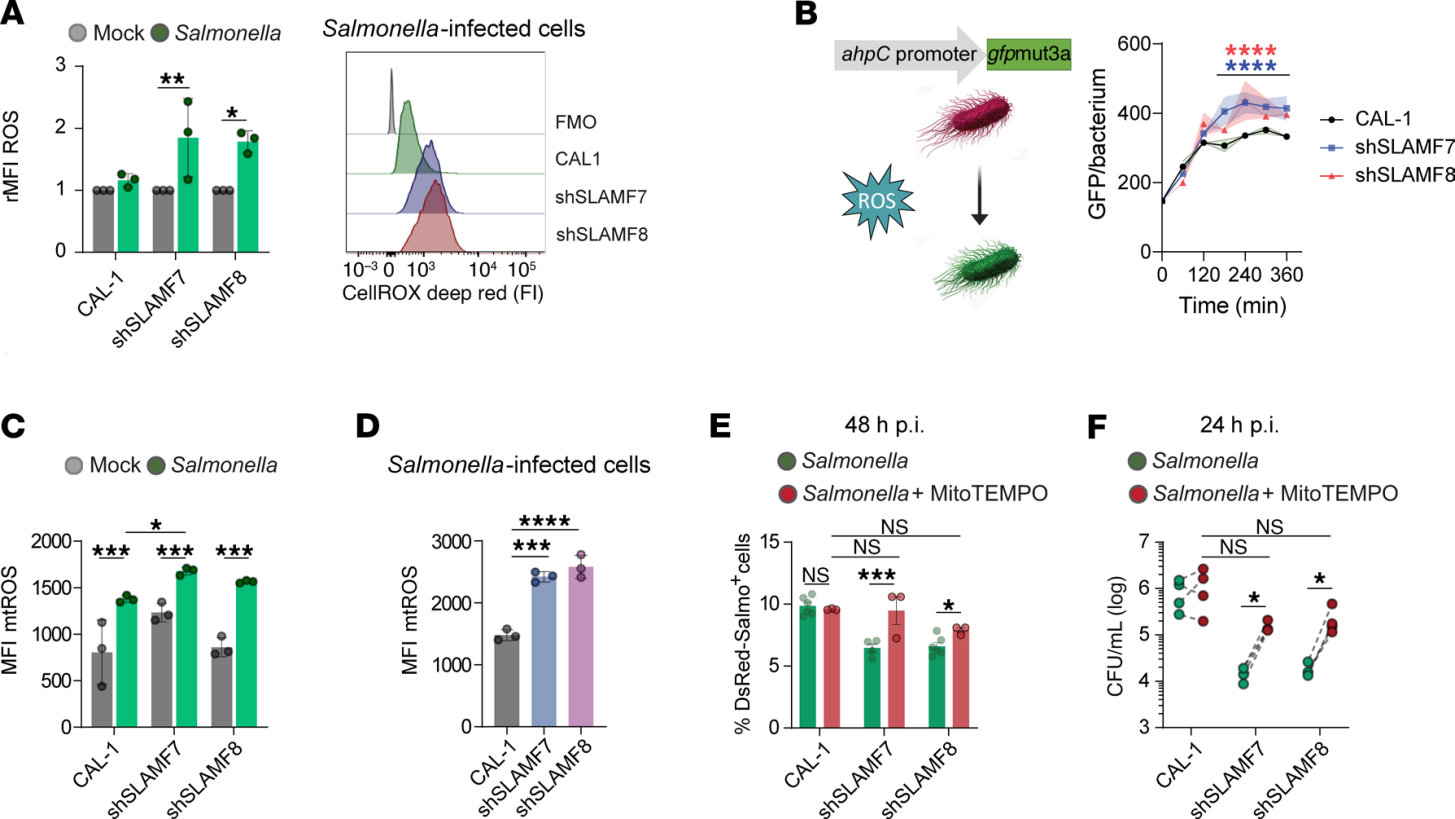
SLAMF7 and SLAMF8 restrict *Salmonella* clearance by limiting mitochondrial ROS accumulation in human pDCs. (**A**) Global cellular ROS in CAL-1 and SLAMF7- or SLAMF8-KD cells loaded with CellROX Deep Red fluorogenic probe, infected with *S*. Typhimurium (green) for 4 hours and analyzed by flow cytometry. Left: Column graphs of the relative MFI (rMFI) of CellROX Deep Red to mock-treated CAL-1 (gray) cells put arbitrarily at 1. Each individual point represents 1 independent experiment. Mean ± SD. *n* = 3. Right: Histograms for total ROS content upon *Salmonella* infection (FMO, fluorescence minus one negative control). (**B**) Oxidative stress detection by intracellular *Salmonella*. CAL-1 and SLAMF-silenced cells were infected with the ROS-sensing mutant *Salmonella* strain carrying the ahpC-gfp (left, scheme). Cells were lysed at different time points p.i., and GFP fluorescence intensity of intracellular bacteria was determined by flow cytometry. Results (right) are representative of *n* = 3. (**C** and **D**) Mitochondrial superoxide detection in CAL-1 and SLAMF-silenced cells infected with DsRed–*S*. Typhimurium for 4 hours using MitoSOX and flow cytometry. (**C**) Column graphs show MitoSOX MFI in the total population. Each individual point represents 1 independent experiment. Mean ± SD. *n* = 3. (**D**) mtROS levels in gated *Salmonella*-infected cells from **C**. (**E** and **F**) After internalization (2 hours p.i.), DsRed-*Salmonella*–infected cells were treated or not with MitoTEMPO (100 μM) and further incubated for the indicated times. *n* = 4 for CAL-1; *n* = 3 for SLAMF-KD. (**E**) Proportion of DsRed-*Salmonella*–infected cells determined at 48 hours p.i. by flow cytometry. Each individual point represents 1 independent experiment. Mean ± SD. (**F**) Intracellular bacterial burden determined by CFU quantification at 24 hours p.i. Each individual point corresponds to 1 independent experiment. (**A**–**F**) Two-way ANOVA followed by Šidák’s multiple-comparison test. *P < 0.05; **P < 0.01; ***P < 0.001; ****P < 0.0001.

Given that SLAMF8 inhibits superoxide production via the NOX2 complex in murine macrophages (30), and that enhanced activation and oxidative burst reported in SLAMF8-deficient macrophages result in augmented *Salmonella* clearance (28), we analyzed the content of total reactive oxygen species (ROS) in CAL-1 and SLAMF7- or SLAMF8-KD cells first loaded with the CellROX Deep Red (Thermo Fisher Scientific) fluorogenic probe and then infected with *Salmonella*. Bacterial infection triggered higher levels of ROS in SLAMF7- or SLAMF8-KD cells as compared with CAL-1 cells (Figure 8A). Then, to monitor the oxidative stress directly sensed by the bacteria, we used a *Salmonella* mutant strain, in which GFP synthesis is upregulated in the presence of exogenous or endogenous H_2_O_2_ (38). As shown in Figure 8B, from 180 minutes p.i. onward, GFP fluorescence was significantly more elevated in bacteria infecting SLAMF7-KD or SLAMF8-KD cells than in CAL-1–infecting bacteria. Silencing SLAMF7 or SLAMF8 in pDCs thus augments the oxidative stress sensed by the bacteria.

Since mitochondrial ROS (mtROS) influence type I IFN–producing capacity, activation, and antigen cross-presentation effectiveness of human pDCs (39, 40), and participate as well in microbial clearance in macrophages (41, 42), we next asked whether the enhanced oxidative stress in SLAMF7- or SLAMF8-KD cells involves mtROS. Specific mitochondrial superoxide detection using MitoSOX (Thermo Fisher Scientific) and flow cytometry revealed that *Salmonella* infection increased mtROS levels in pDCs (Figure 8C), and caused a significantly higher mtROS accumulation in SLAMF7- or SLAMF8-KD cells as compared with CAL-1 cells harboring bacteria (Figure 8D). When mtROS were inhibited in *Salmonella*-infected cells using MitoTEMPO (Sigma-Aldrich), a mitochondria-targeted antioxidant, the percentage of infected CAL-1 cells and intracellular bacterial burden remained stable (Figure 8E), but in SLAMF-KD cells a partial reversion in terms of proportion of DsRed-*Salmonella*^+^ cells and CFU counts occurred (Figure 8F). These results indicate that in human pDCs, SLAMF7 and SLAMF8 help *Salmonella* persistence by limiting excessive mtROS accumulation.

### SLAMF7 and SLAMF8 stimulate the NF-κB, IRF7, and STAT-1 pathways in pDCs.

**Figure 9:**
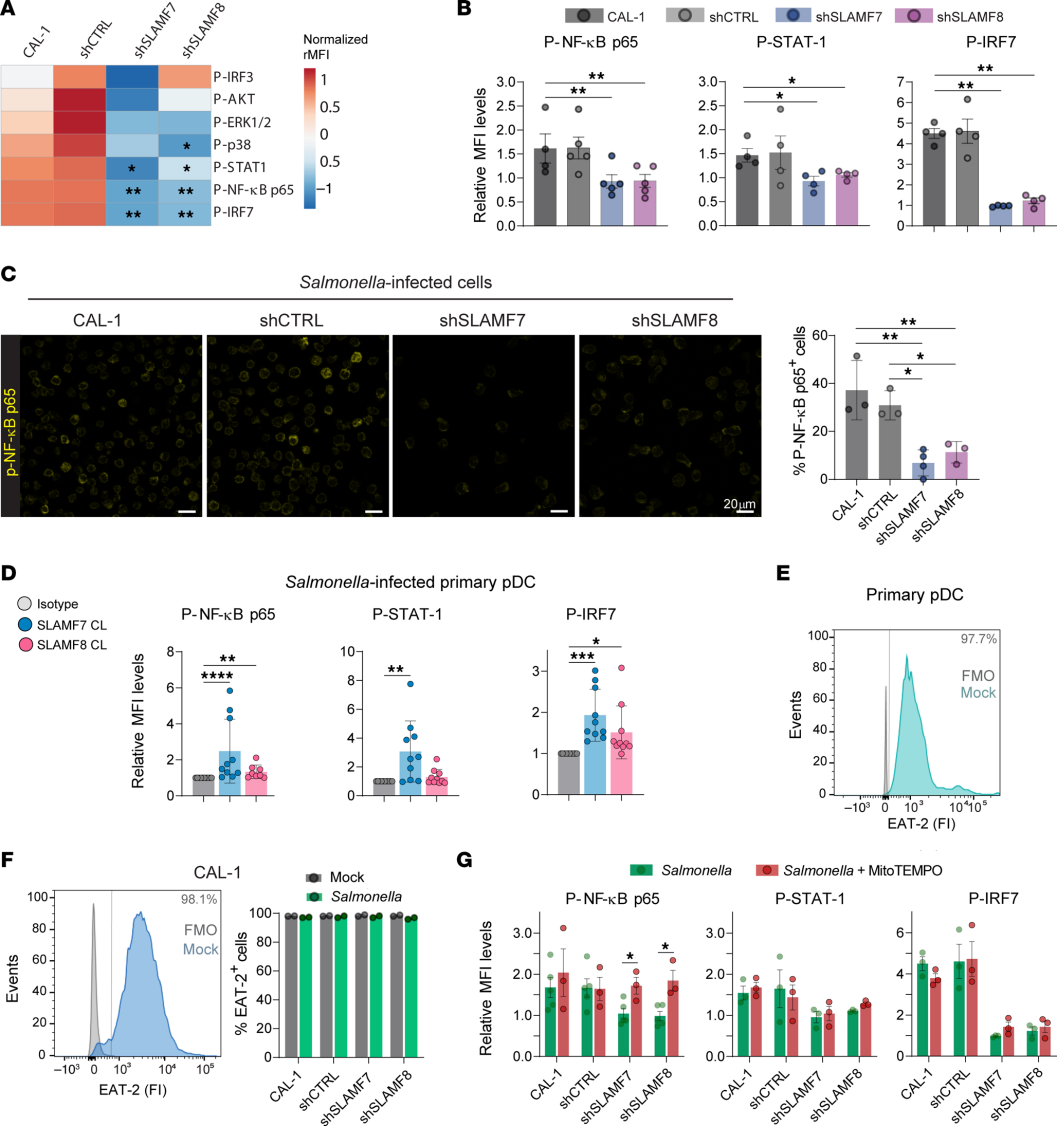
SLAMF7 or SLAMF8 triggers NF-κB, IRF7, and STAT-1 signaling in human pDCs. (**A**–**C**) CAL-1 and stably transduced (shCTRL, shSLAMF7, shSLAMF8) CAL-1 cells were infected with DsRed–*S*. Typhimurium (MOI 25) for 3 hours, and then processed for phospho-flow cytometry or confocal microscopy. (**A**) Heatmap showing MFI levels for p-IRF7, p–NF-κB p65, p–STAT-1, p-p38, p–ERK-1/2, p-AKT, and p-IRF3 of *Salmonella*-infected cells relative to mock condition. Multiple-comparison Kruskal-Wallis test followed by post hoc Dunn’s test. *n* = 4–5. (**B**) Column graphs showing relative MFI of selected phosphorylated proteins in infected cells. Mean ± SD. *n* = 5. (**C**) Confocal images (scale bars: 20 μm) (left) and quantification (right) of p–NF-κB p65 in *Salmonella*-infected CAL-1 and stably transduced CAL-1 cells. *n* = 3. One-way ANOVA followed by Dunnett’s multiple-comparison test. (**D**) Primary human pDCs were infected and analyzed as in **B** but, after 1 hour, SLAMF7 or SLAMF8 were engaged by cross-linking with a specific antibody or its isotype control. One donor per *n*; *n* = 10. (**E**) Flow cytometry histogram of EAT-2 expression (fluorescence intensity [FI]) in primary human pDCs with positivity indicated by a vertical gray bar. One donor per *n*; *n* = 3. (**F**) Left: Flow cytometry histogram of EAT-2 FI in CAL-1 cells at resting state with positivity bar. Right: Percentages of EAT-2^+^ CAL-1 and stably transduced cells, infected (green) or not (gray) as in **B**. *n* = 2. (**G**) CAL-1 and stably transduced CAL-1 cells were infected as in **B**; after 1 hour, washes, and gentamicin addition, MitoTEMPO (100 μM) was added for 2 hours. Then cells were processed for phospho-flow cytometry. Column graphs showing relative MFI of selected phosphorylated proteins in infected cells. Mean ± SD. *n* = 3. Two-way ANOVA followed by Šidák’s multiple-comparison test. Only statistically significant differences are shown. *P < 0.05; **P < 0.01; ***P < 0.001; ****P < 0.0001. No *P* value indicates not significant.

To decipher the intracellular signaling pathways triggered downstream of SLAMF7 and SLAMF8 in human pDCs, we performed phospho-flow cytometry on infected cells. Phosphorylation levels of NF-κB p65, IRF7, and STAT-1 were significantly higher in *Salmonella*-infected CAL-1 or shCTRL cells than in SLAMF7- or SLAMF8-KD cells (Figure 9, A and B). A similar trend was also observed for phosphorylated AKT and p38. Consistently, by confocal microscopy, the percentage of phosphorylated NF-κB p65^+^ cells at 3 hours p.i. was lower when SLAMF7 or SLAMF8 was silenced (Figure 9C). In human primary pDCs cross-linked with specific antibodies, phosphorylation levels of NF-κB p65, IRF7, and STAT-1 were significantly above those observed in the isotype control antibody condition (Figure 9D). SLAMF receptor functions are controlled by the SLAM-associated protein (SAP) family of adaptors, comprising SAP, EAT-2, and ERT, which either block the recruitment of inhibitory phosphatases (like SHP-1 or SHP-2) or directly activate signaling pathways. These interactions dictate the activation or inhibitory nature of the SLAMF receptors upon engagement (43, 44). Here, we found that EAT-2 was highly expressed in primary human pDCs (Figure 9E), as well as in CAL-1 cells, and stable upon infection with *Salmonella* and when SLAMF7 or SLAMF8 was downregulated (Figure 9F). Finally, we examined whether the higher levels of mtROS found in the absence of SLAMF7 or SLAMF8 influence the intracellular signaling initiated in pDCs by *Salmonella* infection. Treatment with MitoTEMPO restored the phosphorylation levels of NF-κB p65 in SLAMF7- or SLAMF8-KD cells at 3 hours p.i. to those found in CAL-1 or shCTRL cells (Figure 9G). In contrast, mtROS pharmacological inhibition did not affect the phosphorylation of STAT-1 or IRF7 (Figure 9G). These data imply that, in *Salmonella*-infected pDCs, moderation of mtROS levels by SLAMF7 or SLAMF8 supports NF-κB activation.

## Discussion

In this work, we resolve the long-standing debate regarding the direct infectibility and activation of human pDCs by bacterial pathogens (45–49). Using two different models of intracellular bacteria leading to acute (*S*. Typhimurium) or chronic (*Brucella* spp.) infection, we demonstrate that these bacteria not only infect pDCs but also control pDC function in a SLAMF7- and SLAMF8-codependent fashion. We found that in pDCs SLAMF7 and SLAMF8 are coregulated at the protein and mRNA levels in steady state and upon infection or TLR7/8 stimulation. This coregulation is consistent with the simultaneous variation of *SLAMF7* and *SLAMF8* mRNA expression in our human blood transcriptomic analyses from a wide range of infectious disease patients, but differs from the sole upregulation of SLAMF7, and not SLAMF8, in PBMCs and monocytes/macrophages of sepsis patients or inflamed synovial tissue (27, 50). Using heat-killed *Salmonella* or purified bacterial RNA, we show that SLAMF7 and SLAMF8 upregulation in pDCs likely depends at least partially on TLR7/8. We further reveal that whereas LPS exposure of isolated primary pDCs (Figure 3C) does not trigger any increase in SLAMF7 and SLAMF8 expression levels in vitro, it does so when pDCs are stimulated with other PBMCs (Figure 3, B and C). This points to the essential role of environmental context (including additional immune and non-immune cells and cytokine secretion along with contact-dependent interactions) in eliciting the full pDC response to infection. A link between intestinal pDC function and enteric serotonergic neurons during *S*. Typhimurium infection has been recently disclosed in the mouse (51); this illustrates the complexity of the environment that also connects the immune and nervous systems to finely tune pDC functions.

Our findings identify human SLAMF7 and SLAMF8 as immunoreceptors that trigger human pDC maturation, activation, and proinflammatory cytokine and type I IFN secretion, and promote as well the survival of intracellular bacteria during infection. This activating function of SLAMF7 and SLAMF8 during *Brucella* infection in pDCs contrasts with the restriction of DC activation driven by the interaction of SLAMF1 with *Brucella* Omp25, which also facilitates bacterium intracellular survival (14) and the negative regulation of proinflammatory cytokine production in macrophages by SLAMF7 upon TLR4 stimulation (50). Besides type I and III IFNs, we found that *Salmonella*- and *Brucella*-infected pDCs secrete TNF-α in a SLAMF7/8-dependent manner, which is coherent with the SLAMF7/8-dependent activation of the NF-κB, STAT-1, and IRF7 pathways that we observed in *Salmonella*-infected primary pDCs or CAL-1 cells. These findings concur with the rapid induction of TNF-α upon SLAMF7 engagement on macrophages, amplifying cell activation through an autocrine loop (27), and with the cell-intrinsic TNF signaling that promotes pDC IFN production during mouse cytomegalovirus infection in vivo (52). Future studies should reveal whether the SLAMF7/8-elicited type I IFN production by pDCs plays a role in the maintenance of immune homeostasis via metabolic reprogramming in brucellosis or salmonellosis, as globally reported for type I IFN during chronic viral infection (53–55).

Regarding the SAP family of adaptors in human pDCs, we found that EAT-2 was expressed in both primary pDCs and WT or KD CAL-1 cells, and stable upon *Salmonella* infection. This shows that while CAL-1 cells do not fully replicate primary pDCs, all our findings concerning SLAMF7/8 (positive regulation of pDC maturation and activation, as well as signaling) are shared by both cell types. It suggests also that in human pDCs EAT-2 may interact with SLAMF7 intracellular tyrosine-based switch motifs (ITSMs) upon infection, evoking the direct SAP-independent association of SLAMF7 with EAT-2 that triggers NK cell activation and cytotoxicity (56–58). It remains possible that, as with tumor cell elimination by macrophages (37), SLAMF7 downstream signaling during infection occurs without adaptor recruitment. SLAMF8, like SLAMF9, lacks ITSMs on its cytoplasmatic tail but can also elicit specific transduction pathways (28, 30, 59) via the interaction with phosphatases, such as SHP-2 (59, 60). Further investigation is needed to unravel pDC SLAMF8 interacting partners, which might also associate with SLAMF7 given their common behavior in these cells upon infection.

In human pDCs, we observed that SLAMF7 or SLAMF8 silencing enhances *Salmonella* clearance, ROS production, and the oxidative stress directly sensed by the bacteria, in agreement with prior work on SLAMF8-deficient mouse macrophages (28). We further showed that the higher levels of mtROS seen in SLAMF7- or SLAMF8-KD pDCs are responsible for this bacterial clearance. This demonstrates that SLAMF7 and SLAMF8 control of mtROS levels facilitates bacteria intracellular survival. The tempering of mtROS is most probably achieved upon engagement of SLAMF7 and SLAMF8 through the PI3K pathway, since we observed less AKT phosphorylation in SLAMF7/8-KD cells. This is in accordance with the reported role of SLAMF8 in macrophages (28). We also found that when SLAMF7 or SLAMF8 is silenced, NF-κB phosphorylation is decreased, but restored to WT levels by the inhibition of mtROS. These data are consistent with the complex role played by the oxidative stress on NF-κB activation (61); they also indicate that the SLAMF7/8-dependent limitation of mtROS indirectly acts as a positive regulatory loop in the activation of NF-κB and pDC function. Overall, while mtROS is known to influence the activation and type I IFN–producing capacity of pDCs (39, 40), to our knowledge this work is the first to describe a limitation of mtROS signaling pathways in pDCs.

Reduction of mtROS, whose levels are downregulated in a SLAMF7/8-dependent fashion during infection (this study), restrains cross-presentation in pDCs and, notably, cross-priming of CD8^+^ T cells (40). In contrast, SLAMF7 or SLAMF8 engagement superactivates pDCs (this study). These findings illustrate the fine-tuning of human pDC inflammatory response to infection orchestrated by SLAMF7 or SLAMF8. Thus, in pDCs, which bridge innate to acquired immunity, SLAMF7/8 signaling may constitute a pivotal mechanism for shaping T cell homeostasis and effector function during bacterial infection, as well as for controlling bacterial survival. In macrophages, key cells of innate immunity, SLAMF8 negatively controls ROS production and bacterial survival in the mouse (28), and LPS-triggered SLAMF7 inhibits murine cell activation (50), whereas SLAMF7 engagement strongly activates human cells (27). These differences highlight the cell- and species-specific response of immune cells to pathogens that may derive from differential adaptor recruitment and context-dependent signaling.

Natural bacterial ligands of SLAMF7 and SLAMF8 remain to be identified; they might correspond to various sorts of microbial entities as known for 3 SLAMF family members — SLAMF1: measles virus, *E*. *coli* OmpC and OmpF proteins (reviewed in ref. 19), and *B*. *abortus* Omp25 (14, 16); SLAMF2: *E*. *coli* FimH protein (19); and SLAMF6: *E*. *coli* OmpC and OmpF proteins (18). However, one has to keep in mind that the existence of a microbial ligand denotes a long coevolution and adaptation of the pathogen to its host(s) and that SLAMF receptors, which behave as self-ligands, such as SLAMF7 or SLAMF8, may have none.

We have shown that bacterial RNA upregulates both SLAMF7 and SLAMF8 in human pDCs, and engagement of SLAMF7/8 receptors positively controls human pDC function. Hence, we propose that SLAMF7 and SLAMF8 act as homotypic interactors that functionally overlap in human pDCs. SLAMF7 and SLAMF8 also crosstalk with TLR7/8 in endosomes in order to contribute to the immune host defense against intracellular bacteria and their byproducts such as bacterial RNA, and at the same time may help intracellular bacteria to establish a chronic infection.

Thus, besides SLAMF9 involved in pDC homeostasis in a full organism (20), SLAMF7 and SLAMF8 emerge as paramount members of the SLAMF receptor family positively regulating pDC response to intracellular bacteria. SLAMF7 is also upregulated in monocytes/macrophages in several types of viral chronic inflammation (27, 62, 63) as well as in myelofibrosis (64) and atherosclerosis (65). Given the consequences of elevated type I IFN secretion in various disorders, the involvement of SLAMF7 and SLAMF8 extends beyond infectious diseases to cancer and autoimmunity (9, 21, 37, 43, 59, 60, 62, 66). Therefore, the concerted role of SLAMF7 and SLAMF8 in orchestrating human pDC responses against bacteria suggests that cell-specific blockade of these receptors may constitute a promising therapy to abate pDC activation and hyperproduction of type I IFN during infection, as well as in autoimmune disorders and cancer.

## Methods

### Sex as a biological variable.

All patient cohorts used in this study were sex-matched with healthy control individuals, and samples were obtained from both men and women. Percentage of men and women for each cohort is shown in Supplemental Tables 1–10 and Supplemental Figure 1, G and H. For the rest of our study that involved PBMCs (including primary pDCs prepared from PBMCs), sex was not considered as a biological variable. As regards CAL-1 cells, this cell line was established 20 years ago from the peripheral blood of a man with blastic natural killer lymphoma in leukemic phase after chemotherapy, and was categorized as CD4^+^CD56^+^ pDCs (67).

### Cell culture and treatments.

Healthy human blood and buffy coats were obtained by leukapheresis (Etablissement Français du Sang, Marseille, France). PBMCs were isolated by centrifugation over Ficoll-Hypaque (17144003, Cytiva, Sigma-Aldrich) and cultured (1 × 10^6^ cells/mL) in flat-bottom 48-well plates with RPMI 1640 (21875034, Gibco, Thermo Fisher Scientific) supplemented with l-glutamine (25030024, Gibco, Thermo Fisher Scientific) and 10% fetal bovine serum (FBS) (P30-3306, PAN-Biotech Dutscher). Purification of primary pDCs was performed using the Plasmacytoid Dendritic Cell Isolation Kit II (130-097-415, Miltenyi Biotec), reaching greater than 93% purity and high yields (between 1 and 2 million viable pDCs depending on the volume of blood received per human donor. For cross-linking experiments, primary pDCs were infected with DsRed–*Salmonella* Typhimurium for 1 hour. After washes and gentamicin treatment as described below, cells were then moved into a fresh SLAMF7 or SLAMF8 (or isotype control) monoclonal antibody–cross-linked plate. Soluble SLAMF7 or SLAMF8 (or isotype control) antibodies were added for 1 hour. Then cells were washed twice, and secondary antibodies were added for the remaining time.

CAL-1 cells (a gift from P. Pierre, CIML) (67) were grown in RPMI 1640, 10% FBS, 2 mM l-glutamine, 1× non-essential amino acids (11140050, Gibco, Thermo Fisher Scientific), 10 mM HEPES (15630080, Gibco, Thermo Fisher Scientific), and 1 mM sodium pyruvate (11360039, Gibco, Thermo Fisher Scientific). For experiments, cells were plated in 24-well plates at 4 × 10^5^ cells/mL, 1 mL/well, in complete medium with 2% FBS. All cell lines were mycoplasma-free as determined with the MycoAlert Mycoplasma Detection Kit (LT07-318, Lonza).

Cells were infected or stimulated with bacterial components or TLR ligands as specified in the figure legends and analyzed by flow cytometry. Highly purified TLR ligands were used: CpG (tlrl-m362, InvivoGen), poly(I:C) (tlrl-pic, InvivoGen), PAM3 (tlrl-pms, InvivoGen), flagellin (tlrl-stfla, InvivoGen), *E*. *coli* LPS serotype O55:B5 (ALX-581-013-L002, Enzo Life Sciences), R848 (tlrl-r848-1, InvivoGen), and *Brucella melitensis* (Bm) LPS (68). Outer membrane vesicles, *Brucella abortus* (Ba) OMV WT and Omp25, were gifts from I. Moriyón (Universidad de Navarra, Pamplona, Spain) (16). Bacterial RNA was purified from the *E*. *coli* strain DH10B or from the *Salmonella* Typhimurium WT strain (see below) with the Direct-zol RNA Miniprep Kit (ZR2070T, Zymo Research Ozyme) following the manufacturer’s instructions. RNA stimulation was performed in the presence of Lipofectamine (Thermo Fisher Scientific) to facilitate access to endosomal sensors and protect RNA from degradation. When specified, MitoTEMPO (100 μM; SML0737, Sigma-Aldrich) was added to cell culture.

### Mutant CAL-1 cell generation.

Specific knockdown CAL-1 cells were generated by transduction of lentiviral particles containing short hairpin RNAs (shRNAs) targeting SLAMF7 and SLAMF8. Briefly, specific shRNA-expressing plasmids (pGFP-C-shLentiSLAMF7 and pGFP-C-shLentiSLAMF7, Origene) with pCMV-VSV-G envelope plasmid and pCMV-dR8.91 Gag and polymerase expression plasmid (gifts from M. Negroni, IBMC Strasbourg, France) were transfected into HEK293T cells by the calcium phosphate method. Two days after transfection, the viral supernatant was harvested, filtered through a 0.45 μm filter, and concentrated on Amicon Ultra-15 centrifugal filters (C7715, Merck). The concentrated viral particles were added to CAL-1 cells together with lentiBLAST Premium transduction enhancer (1 μL; LBPX500, OZ Biosciences) and centrifuged for 90 minutes at 800*g*. Cells were incubated for 48 hours, after which the medium was replaced with fresh complete medium. Puromycin selection (0.5 μg/mL; A1113803, Thermo Fisher Scientific) was then applied to select for cells that had stably integrated the lentiviral construct. Monoclonal cell lines were obtained by limiting dilution. The selected cells were further characterized by flow cytometry and quantitative reverse transcriptase PCR to confirm knockdown efficiency and GFP expression. Control cells were generated in parallel using lentiviral particles containing non-targeting shRNA. Transduced CAL-1 clones were maintained in complete medium supplemented with puromycin (0.2 μg/mL).

### Bacterial strains and infection.

The following *Salmonella* Typhimurium and isogenic strains were used in this study: *Salmonella enterica* subsp. *enterica* serovar Typhimurium (*S*. Typhimurium strain 12023, laboratory stock); *S*. Typhimurium DsRed strain (69); and *S*. Typhimurium ROS sensing strain, which contains an OxyR-dependent promoter fused to the *gfp*mut3a gene and lacks four H_2_O_2_-degrading enzymes (*katE*, *katG*, *katN*, *ahpCF*) (39). Strains were cultured in Luria broth medium (Difco, Thermo Fisher Scientific) at 37°C, and ampicillin (50 μg/mL), kanamycin (50 μg/mL), or chloramphenicol (50 μg/mL) was added when required.

Fluorescent *Brucella* (bIN#1559 for *B*. *abortus* strain 2308; bIN#1502 for *B*. *melitensis* strain 16M) were obtained by transformation with a pMR10-based plasmid encoding mCherry (70). They were cultured on tryptic soy agar plates containing kanamycin (25 μg/mL) at 37°C. All *Brucella* were kept, grown, and used under strict biosafety containment conditions throughout the experiments in the BSL3 facility of Virulence Bactérienne et Infections Chroniques, Nîmes, France.

### S.

Typhimurium at a multiplicity of infection (MOI) of 25:1 or *Brucella* spp. at an MOI of 5,000:1 were used to infect human primary pDCs or CAL-1 cells. Bacteria were plated onto cells in 100 μL and left for 1 hour at 37°C with 5% CO_2_. After 2 washes with medium, cells were incubated for 1 hour in medium containing 50 μg/mL gentamicin to kill extracellular bacteria. Thereafter, antibiotics concentration was decreased to 5 μg/mL.

### Cytometry.

Cells were harvested and stained for 30 minutes at 4°C with the primary antibody mix. Then cells were washed in PBS with 2% of FCS and incubated with the secondary antibody mix for 30 minutes at 4°C. When needed, a third step of antibody incubation was added for staining with coupled antibodies. After washing, cells were stained with LIVE/DEAD Fixable Blue Dead Cell Stain (L34961, Invitrogen, Thermo Fisher Scientific) for 10 minutes at 20°C. Cells were then fixed for 10 minutes in 3.2% paraformaldehyde (PFA) at 20°C or analyzed immediately. Antibodies were the following: SLAMF1, clone A12(7D4), 306302; SLAMF7/CD319/CRACC, clone 162.1, 331802; mouse IgG1, clone RMG1-1, 40615; mouse IgG2b κ, clone MPC-11, 400342; CD16, clone 3G8, 302026; HLA-DR, clone L243, 307640; CD3, clone SK7, 344823; XCR1, clone S13946E, 372627; CD141, clone M80, 344108; CD1c, clone L161, 331533; CD11c, clone Bu15, 337247; and CD16, clone 3G8, 302026, were from BioLegend. SLAMF8, clone 250014, MA523947; PD-L1/CD274, clone MIH1, 46598342; CD274/PD-L1, clone MIH1, 46-5983-42; and CD303, clone 201A, eBioscience 11-9818-42, were from Thermo Fisher Scientific. CD123, clone 9F5, 551065; CD14, clone MΦP9, 560180; CD3, clone SK7, 563798; CD56, clone B159, 555516; CD80, clone 307.4, 557227; CD86, clone FUN-1, 563412; AXL, clone 108724, 747866; and Siglec-6, clone 767329, 747916, were from BD Biosciences. CD4, clone SK3, SKU R7-20074; CD8, clone SK1, SKU R7-20036; and CD19, clone SJ25C1, SKU R7-20274, were from Cytek Biosciences. EAT2/SH2D1B (clone 2B4, catalog SAB1401970) was from Sigma-Aldrich and the mouse IgG2a κ isotype antibody (clone eBM2a, catalog 14-4724-82) for the fluorescence minus one control (FMO) from eBioscience/Thermo Fisher Scientific. EAT-2 and phospho-flow staining was performed by fixing of the cells with 3.2% PFA for 10 minutes at 20°C, followed by permeabilization with cold methanol for 15 minutes. After washing, intracellular staining with antibodies for EAT-2, phospho-proteins (p–NF-κB p65 [Ser536] clone 93H1, 3033; p-Stat1 [Tyr701] clone D4A7, 7649; p-IRF7 [Ser477] clone D7E1W, 42016; p-IRF3 [Ser396], 29047; p-AKT [Thr308], 9275; p–p38 MAPK [Thr180/Tyr182], 9211; p–p44/42 MAPK [Erk1/2] (Thr202/Tyr204) clone D13.14.4E, 4370; all from Cell Signaling Technology) or isotype controls was done in 1× PBS/2% BSA. Flow cytometry was performed using a FACS Aurora (Cytek) for PBMCs or a Fortessa (BD Biosciences) for primary and pDC cell lines and *Salmonella*, and data were analyzed with Flow Jo v9.9.4 software. Surface staining gating relied on the examination of mock cell and isotype control antibody staining. In PBMCs, the various cell subsets were identified by spectral cytometry, based on SSC/FSC, singlets, and viable cells after staining with LIVE/DEAD Fixable Blue Dead Cell Stain (L34961, Invitrogen, Thermo Fisher Scientific) as the following: CD4^+^ T cells (HLA-DR^–^CD14^–^CD19^+^CD3^+^CD4^+^), CD8^+^ T cells (HLA-DR^–^CD14^–^CD19^+^CD3^+^CD4^+^), B cells (CD14^–^CD3^–^CD19^+^), CD56^–^CD16^+^ NK cells (CD3^–^CD19^–^CD14^–^CD56^–^CD16^+^), CD56^dim^CD16^+^ NK cells (CD3^–^CD19^–^CD14^–^CD56^dim^CD16^+^), CD56^bright^CD16^–^ NK cells (CD3^–^CD19^–^CD14^–^CD56^bright^CD16^+^), classical monocytes (CD3^–^CD19^–^CD56^–^CD14^++^CD16^+^), intermediate monocytes (CD3^–^CD19^–^CD56^–^CD14^++^CD16^–^), non-classical monocytes (CD3^–^CD19^–^CD56^–^CD14^+^CD16^++^), cDC1 cells (CD3^–^CD19^–^CD56^–^HLA-DR^+^CD14^–^CD141^+^XCR1^+^CD11c^+^), cDC2 cells (CD3^–^CD19^–^CD56^–^HLA-DR^+^CD14^–^CD1c^+^CD11c^+^), pDC-like cells (CD3^–^CD19^–^CD56^–^HLA-DR^+^AXL^+^Siglec-6^+^), and pDCs (CD3^–^CD19^–^CD56^–^HLA-DR^+^CD11c^–^CD123^+^CD303^+^). Spectral cytometry was performed using an Aurora machine (Cytek), and data were analyzed with SpectroFlo software (Cytek).

### Transcriptome dataset selection and population study.

We first browsed the Gene Expression Omnibus (GEO) repository using the following keywords and expressions: [(virus) or (bacteria) or (parasite) or (infection) or (parasite) or (pathogen)] and [(transcriptomics) or (RNA-Seq) or (microarray) or (expression) or (transcriptome)] and [(blood) or (whole blood)]. All potentially relevant datasets were further evaluated in detail. The eligibility criteria included (a) publicly available transcriptome data; (b) detailed sample information; (c) detailed protocol information; (d) inclusion of healthy donor controls; and (e) same sample source — whole blood.

For detailed human studies on salmonellosis, a first cohort of patients was referenced as GSE113866 (33), and a second cohort as GSE69529 (34).

Brucellosis patients were handled at the University Clinic for Infectious Diseases and Febrile Conditions, Skopje, spanning the years 2007 to 2015. The study encompassed a total of 108 patients, categorized as follows: acute treated (*n* = 54; 14.54% women; age range 4–73 years with median age 43), acute with relapse (*n* = 6; 16.66% women; age range 17–59 years with median age 39), chronic (*n* = 12; 18.18% women; age range 28–70 years with median age 38), and healthy donors (*n* = 36; similar median age and sex ratio). Informed consent was obtained from the patients and healthy donors enrolled in the study, based on routine diagnostic blood collection. RNA sequencing was conducted using a protocol established by the Benaroya Research Institute, Seattle, Washington, USA. Serum samples from these patients, as well as healthy donor control blood, were stored at –80°C for subsequent cytokine/chemokine analysis. The original RNA-Seq datasets were deposited in the GEO repository under accession number GSE69597 (25, 26). The diagnosis of brucellosis was based on clinical signs and symptoms relevant to this disease (fever, sweating, malaise, arthralgia, hepatomegaly, splenomegaly, and focal disease signs), and validated by a qualitative positive rose bengal test and a Brucellacapt assay (Vircell) of greater than 1/320.

For each patient cohort, demographic and clinical features are summarized in a specific Supplemental Table (Supplemental Table 1, COVID-19; Supplemental Table 2, flu; Supplemental Table 3, malaria; Supplemental Table 4, Lyme disease; Supplemental Table 5, tuberculosis; Supplemental Table 6, streptococcal infection; Supplemental Table 7, streptococcal pharyngitis; Supplemental Table 8, brucellosis; Supplemental Table 9, salmonellosis cohort 1; Supplemental Table 10, salmonellosis cohort 2). No reporting on race or ethnicity was done.

### Microarray differential expression analysis.

Data were log_2_-transformed, and force normalization was applied. GEOquery and limma (Linear Models for Microarray Analysis) were used for differential expression analysis and R. Benjamini and Hochberg false discovery rate method was used for multiple-testing corrections.

### RNA-Seq differential expression analysis.

Pseudo-aligned and pre-processed RNA-Seq data were downloaded. Samples with more than 70% of total genes with 0 sequence reads were deemed as very low-quality and filtered out. Normalization, batch effect correction, and differential expression were performed with R package DESeq2 v1.28.1 (71). Wilcoxon’s rank sum test was done to determine statistical differences between categorical groups.

All other experimental procedures are described in Supplemental Methods.

### Statistics.

Statistical analyses were carried out using R or GraphPad Prism v9 and v10 software. Shapiro-Wilk test was used to assess the normality of data distribution. Analysis of variance (ANOVA) followed by Dunnett’s multiple-comparison test, Kruskal-Wallis test followed by post hoc Dunn’s test, or 2-way ANOVA with Šidák’s multiple-comparison test was used as indicated in the figure legends. Mann-Whitney *U* test and Wilcoxon’s rank sum test were used for the analysis of unpaired and paired samples, respectively. Correlations were calculated using the non-parametric Spearman’s correlation test. Differences between values were considered significant at *P* less than 0.05. All experiments were performed at least 3 times unless otherwise indicated.

### Study approval.

The brucellosis study in humans was approved by the Ethics Committee of the Medical Faculty in Skopje, Republic of North Macedonia (authorization 03-7670/2). Laboratory data were obtained during routine diagnostic procedures, and written consent was given for all patients and healthy donors enrolled.

### Data availability.

Brucellosis patients’ original RNA-Seq data were deposited in GEO (GEO689 NCBI-NIH; https://www.ncbi.nlm.nih.gov/geo/query/acc.cgi?acc=GSE69597). All data associated with the main article and supplemental material are provided in the Supporting Data Values file. Illustration in the graphical abstract was created using BioRender (biorender.com).

## Author contributions

JPG and S Mémet conceptualized and supervised the study. JMP, AK, LG, S Méresse, JPG, and S Mémet designed research. JMP, AK, LG, S Méresse, JPG, and S Mémet contributed to methodology. JMP, AK, VAG, SH, and S Mémet performed experiments. JMP, AK, SH, MB, JS, JPG, and S Mémet analyzed data. LG and S Méresse provided reagents. MB and JS dealt with human sample collection. JMP and S Mémet wrote the original draft. JMP, AK, S Méresse, JPG, and S Mémet edited the manuscript. JPG and S Mémet administrated the project and funding. All authors read and agreed to the published version of the article.

## Supplementary Material

Supplemental data

Supporting data values

## Version 1 04/15/2025

Electronic publication

## Version 2 09/05/2025

Corrected text per corrigendum

## References

[1] Reizis B (2019). Plasmacytoid dendritic cells: development, regulation, and function. Immunity.

[2] Bencze D (2021). Type I interferon production of plasmacytoid dendritic cells under control. Int J Mol Sci.

[3] Kotov DI (2023). Early cellular mechanisms of type I interferon-driven susceptibility to tuberculosis. Cell.

[4] Rahman T (2019). Plasmacytoid dendritic cells provide protection against bacterial-induced colitis. Front Immunol.

[5] Takagi H (2011). Plasmacytoid dendritic cells are crucial for the initiation of inflammation and T cell immunity in vivo. Immunity.

[6] Wu V (2016). Plasmacytoid dendritic cell-derived IFNα modulates Th17 differentiation during early Bordetella pertussis infection in mice. Mucosal Immunol.

[7] Ang DK (2010). Cutting edge: Pulmonary Legionella pneumophila is controlled by plasmacytoid dendritic cells but not type I IFN. J Immunol.

[8] Lippitsch A (2019). Plasmacytoid dendritic cell depletion modifies FoxP3+ T cell homeostasis and the clinical course of bacterial pneumonia in mice. J Leukoc Biol.

[9] Farhangnia P (2023). SLAM-family receptors come of age as a potential molecular target in cancer immunotherapy. Front Immunol.

[10] Ostrowski SR (2005). 2B4 expression on natural killer cells increases in HIV-1 infected patients followed prospectively during highly active antiretroviral therapy. Clin Exp Immunol.

[11] Ward J (2007). HIV modulates the expression of ligands important in triggering natural killer cell cytotoxic responses on infected primary T-cell blasts. Blood.

[12] Berger SB (2010). SLAM is a microbial sensor that regulates bacterial phagosome functions in macrophages. Nat Immunol.

[13] Yurchenko M (2018). SLAMF1 is required for TLR4-mediated TRAM-TRIF-dependent signaling in human macrophages. J Cell Biol.

[14] Degos C (2020). Omp25-dependent engagement of SLAMF1 by *Brucella abortus* in dendritic cells limits acute inflammation and favours bacterial persistence in vivo. Cell Microbiol.

[15] Pellegrini JM (2021). Neutrophil autophagy during human active tuberculosis is modulated by SLAMF1. Autophagy.

[16] Hysenaj L (2023). CD150-dependent hematopoietic stem cell sensing of Brucella instructs myeloid commitment. J Exp Med.

[17] Baorto DM (1997). Survival of FimH-expressing enterobacteria in macrophages relies on glycolipid traffic. Nature.

[18] van Driel B (2015). The cell surface receptor Slamf6 modulates innate immune responses during Citrobacter rodentium-induced colitis. Int Immunol.

[19] van Driel BJ (2016). Responses to microbial challenges by SLAMF receptors. Front Immunol.

[20] Sever L (2019). SLAMF9 regulates pDC homeostasis and function in health and disease. Proc Natl Acad Sci U S A.

[21] Hagberg N (2013). Systemic lupus erythematosus immune complexes increase the expression of SLAM family members CD319 (CRACC) and CD229 (LY-9) on plasmacytoid dendritic cells and CD319 on CD56(dim) NK cells. J Immunol.

[22] Coburn B (2007). Salmonella, the host and disease: a brief review. Immunol Cell Biol.

[23] González-Espinoza G (2021). *Brucella*: reservoirs and niches in animals and humans. Pathogens.

[24] Pellegrini JM (2022). Immunosuppressive mechanisms in brucellosis in light of chronic bacterial diseases. Microorganisms.

[25] https://www.ncbi.nlm.nih.gov/geo/query/acc.cgi?acc=GSE69597.

[26] Dufort MJ (2016). Hepatocytes as immunological agents. J Immunol.

[27] Simmons DP (2022). SLAMF7 engagement superactivates macrophages in acute and chronic inflammation. Sci Immunol.

[28] Romero-Pinedo S (2022). SLAMF8 downregulates mouse macrophage microbicidal mechanisms *via* PI3K pathways. Front Immunol.

[29] Wang G (2015). Migration of myeloid cells during inflammation is differentially regulated by the cell surface receptors Slamf1 and Slamf8. PLoS One.

[30] Wang G (2012). Cutting edge: Slamf8 is a negative regulator of Nox2 activity in macrophages. J Immunol.

[31] Chaudhary A (2018). β-Barrel outer membrane proteins suppress mTORC2 activation and induce autophagic responses. Sci Signal.

[32] Zhang Y (2023). SLAMF8, a potential new immune checkpoint molecule, is associated with the prognosis of colorectal cancer. Transl Oncol.

[33] Blohmke CJ (2019). Diagnostic host gene signature for distinguishing enteric fever from other febrile diseases. EMBO Mol Med.

[34] DeBerg HA (2018). Shared and organism-specific host responses to childhood diarrheal diseases revealed by whole blood transcript profiling. PLoS One.

[35] Sander LE (2011). Detection of prokaryotic mRNA signifies microbial viability and promotes immunity. Nature.

[36] Alculumbre SG (2018). Diversification of human plasmacytoid predendritic cells in response to a single stimulus. Nat Immunol.

[37] Chen J (2017). SLAMF7 is critical for phagocytosis of haematopoietic tumour cells via Mac-1 integrin. Nature.

[38] Aussel L (2011). Salmonella detoxifying enzymes are sufficient to cope with the host oxidative burst. Mol Microbiol.

[39] Agod Z (2017). Regulation of type I interferon responses by mitochondria-derived reactive oxygen species in plasmacytoid dendritic cells. Redox Biol.

[40] Oberkampf M (2018). Mitochondrial reactive oxygen species regulate the induction of CD8^+^ T cells by plasmacytoid dendritic cells. Nat Commun.

[41] West AP (2011). TLR signalling augments macrophage bactericidal activity through mitochondrial ROS. Nature.

[42] Lopez-Perez W (2021). TAK1 inhibition elicits mitochondrial ROS to block intracellular bacterial colonization. Proc Natl Acad Sci U S A.

[43] Wu N, Veillette A (2016). SLAM family receptors in normal immunity and immune pathologies. Curr Opin Immunol.

[44] Yigit B (2019). SLAMF6 in health and disease: implications for therapeutic targeting. Clin Immunol.

[45] Piccioli D (2009). Human plasmacytoid dendritic cells are unresponsive to bacterial stimulation and require a novel type of cooperation with myeloid dendritic cells for maturation. Blood.

[46] Parcina M (2008). Staphylococcus aureus-induced plasmacytoid dendritic cell activation is based on an IgG-mediated memory response. J Immunol.

[47] Bekeredjian-Ding I (2014). Plasmacytoid dendritic cells: neglected regulators of the immune response to staphylococcus aureus. Front Immunol.

[48] Michea P (2013). Epithelial control of the human pDC response to extracellular bacteria. Eur J Immunol.

[49] Raieli S (2019). TLR1/2 orchestrate human plasmacytoid predendritic cell response to gram+ bacteria. PLoS Biol.

[50] Wu Y (2023). SLAMF7 regulates the inflammatory response in macrophages during polymicrobial sepsis. J Clin Invest.

[51] Zhang H (2024). Mouse enteric neurons control intestinal plasmacytoid dendritic cell function via serotonin-HTR7 signaling. Nat Commun.

[52] Abbas A (2020). The activation trajectory of plasmacytoid dendritic cells in vivo during a viral infection. Nat Immunol.

[53] Vastag L (2011). Divergent effects of human cytomegalovirus and herpes simplex virus-1 on cellular metabolism. PLoS Pathog.

[54] Isringhausen S (2021). Chronic viral infections persistently alter marrow stroma and impair hematopoietic stem cell fitness. J Exp Med.

[55] Mackelprang RD (2023). Upregulation of IFN-stimulated genes persists beyond the transitory broad immunologic changes of acute HIV-1 infection. iScience.

[56] Tassi I, Colonna M (2005). The cytotoxicity receptor CRACC (CS-1) recruits EAT-2 and activates the PI3K and phospholipase Cgamma signaling pathways in human NK cells. J Immunol.

[57] Bouchon A (2001). Activation of NK cell-mediated cytotoxicity by a SAP-independent receptor of the CD2 family. J Immunol.

[58] Cruz-Munoz ME (2009). Influence of CRACC, a SLAM family receptor coupled to the adaptor EAT-2, on natural killer cell function. Nat Immunol.

[59] Sugimoto A (2020). SLAM family member 8 is expressed in and enhances the growth of anaplastic large cell lymphoma. Sci Rep.

[60] Sugimoto A (2018). SLAM family member 8 is involved in oncogenic KIT-mediated signalling in human mastocytosis. Exp Dermatol.

[61] Morgan MJ, Liu ZG (2011). Crosstalk of reactive oxygen species and NF-κB signaling. Cell Res.

[62] O’Connell P (2019). SLAMF7 is a critical negative regulator of IFN-α-mediated CXCL10 production in chronic HIV infection. J Immunol.

[63] Zander R (2022). Delineating the transcriptional landscape and clonal diversity of virus-specific CD4^+^ T cells during chronic viral infection. Elife.

[64] Maekawa T (2019). Increased SLAMF7^high^ monocytes in myelofibrosis patients harboring *JAK2*V617F provide a therapeutic target of elotuzumab. Blood.

[65] Xia Z (2018). Integrated DNA methylation and gene expression analysis identifies SLAMF7 as a key regulator of atherosclerosis. Aging (Albany NY).

[66] Zheng Y (2023). Role of signaling lymphocytic activation molecule family of receptors in the pathogenesis of rheumatoid arthritis: insights and application. Front Pharmacol.

[67] Maeda T (2005). A novel plasmacytoid dendritic cell line, CAL-1, established from a patient with blastic natural killer cell lymphoma. Int J Hematol.

[68] Zhao Y (2018). Immunomodulatory properties of Brucella melitensis lipopolysaccharide determinants on mouse dendritic cells in vitro and in vivo. Virulence.

[69] Moest T (2018). Contribution of bacterial effectors and host proteins to the composition and function of Salmonella-induced tubules. Cell Microbiol.

[70] Garcia-Mendez KB (2019). Infection by Brucella melitensis or Brucella papionis modifies essential physiological functions of human trophoblasts. Cell Microbiol.

[71] Love MI (2014). Moderated estimation of fold change and dispersion for RNA-seq data with DESeq2. Genome Biol.

