# Corrigendum to SLAMF7 and SLAMF8 receptors shape human plasmacytoid dendritic cell responses to intracellular bacteria

**DOI:** 10.1172/JCI198064

**Published:** 2025-09-02

**Authors:** Joaquín Miguel Pellegrini, Anne Keriel, Laurent Gorvel, Sean Hanniffy, Vilma Arce-Gorvel, Mile Bosilkovski, Javier Solera, Stéphane Méresse, Sylvie Mémet, Jean-Pierre Gorvel

Original citation: *J Clin Invest*. 2025;135(8):e182467. https://doi.org/10.1172/JCI182467

Citation for this corrigendum: *J Clin Invest*. 2025;135(17):e198064. https://doi.org/10.1172/JCI198064

The authors recently became aware that in the Methods of the original article, the references for the CD56 and EAT2 antibodies were incorrect. In addition, the description of the primary pDC purification and cytometry staining procedures lacked clarity. The corrected sentences are provided below. The HTML and PDF versions of the paper have been updated.

Purification of primary pDCs was performed using the Plasmacytoid Dendritic Cell Isolation Kit II (130-097-415, Miltenyi Biotec), reaching greater than 93% purity and high yields (between 1 and 2 million viable pDCs depending on the volume of blood received per human donor).

CD123, clone 9F5, 551065; CD14, clone MΦP9, 560180; CD3, clone SK7, 563798; CD56, clone B159, catalog 555516; CD80, clone 307.4, 557227; CD86, clone FUN-1, 563412; AXL, clone 108724, 747866; and Siglec-6, clone 767329, 747916, were from BD Biosciences.

EAT2/SH2D1B (clone 2B4, catalog SAB1401970) was from Sigma-Aldrich and the mouse IgG2a κ isotype antibody (clone eBM2a, catalog 14-4724-82) for the fluorescence minus one control (FMO) from eBioscience/Thermo Fisher Scientific. EAT-2 and phospho-flow staining was performed by fixing of the cells with 3.2% PFA for 10 minutes at 20°C, followed by permeabilization with cold methanol for 15 minutes. After washing, intracellular staining with antibodies for EAT-2, phospho-proteins (p–NF-κB p65 [Ser536] clone 93H1, 3033; p-Stat1 [Tyr701] clone D4A7, 7649; p-IRF7 [Ser477] clone D7E1W, 42016; p-IRF3 [Ser396], 29047; p-AKT [Thr308], 9275; p–p38 MAPK [Thr180/Tyr182], 9211; p–p44/42 MAPK [Erk1/2] (Thr202/Tyr204) clone D13.14.4E, 4370; all from Cell Signaling Technology) or isotype controls was done in 1× PBS/2% BSA.

The authors regret the errors and are grateful to Árpád Lányi and Kitti Pázmándi (University of Debrecen, Hungary) for alerting them to the issues.

## Version 1 09/02/2025

Electronic publication

## Version 2 09/05/2025

Corrected source for CD56

