# Supplementary material for: SLAMF7 and SLAMF8 receptors shape human plasmacytoid dendritic cell responses to intracellular bacteria
Source: J Clin Invest. 2025 Apr 15;135(8):e182467. doi: 10.1172/JCI182467 (PMC11996910; doi:10.1172/JCI182467)
Supplement: Supplemental data [file jci-135-182467-s010.pdf]

SLAMF7 and SLAMF8 receptors shape human plasmacytoid dendritic cell responses to intracellular bacteria

Pellegrini et al., 2025

List of supplemental material:

Supplemental Figure 1 (related to Figures 1 and 2). Upregulation of SLAMF7 and 8 at the mRNA and protein levels in several diseases and correlation with brucellosis and salmonellosis.

Supplemental Figure 2 (related to Figures 3 and 4). Inducibility of SLAMF7, SLAMF8 and SLAMF1 in human pDC, and infectability of CAL-1 cells.

Supplemental Figure 3 (related to Figure 5). Generation and analysis of SLAMF7- or SLAMF8-silenced CAL-1 cells.

Supplemental table 1. COVID-19 cohort features.

Supplemental table 2. Flu cohort features.

Supplemental table 3. Malaria cohort features.

Supplemental table 4: Lyme disease cohort features.

Supplemental table 5. Tuberculosis cohort features.

Supplemental table 6. Staphylococcal Infection cohort features.

Supplemental table 7. Streptococcal Pharyngitis cohort features.

Supplemental table 8. Brucellosis cohort features.

Supplemental table 9. Salmonellosis cohort 1 features.

Supplemental table 10. Salmonellosis cohort 2 features.

Supplemental methods

Supplemental Figure 1 (related to Figures 1 and 2)

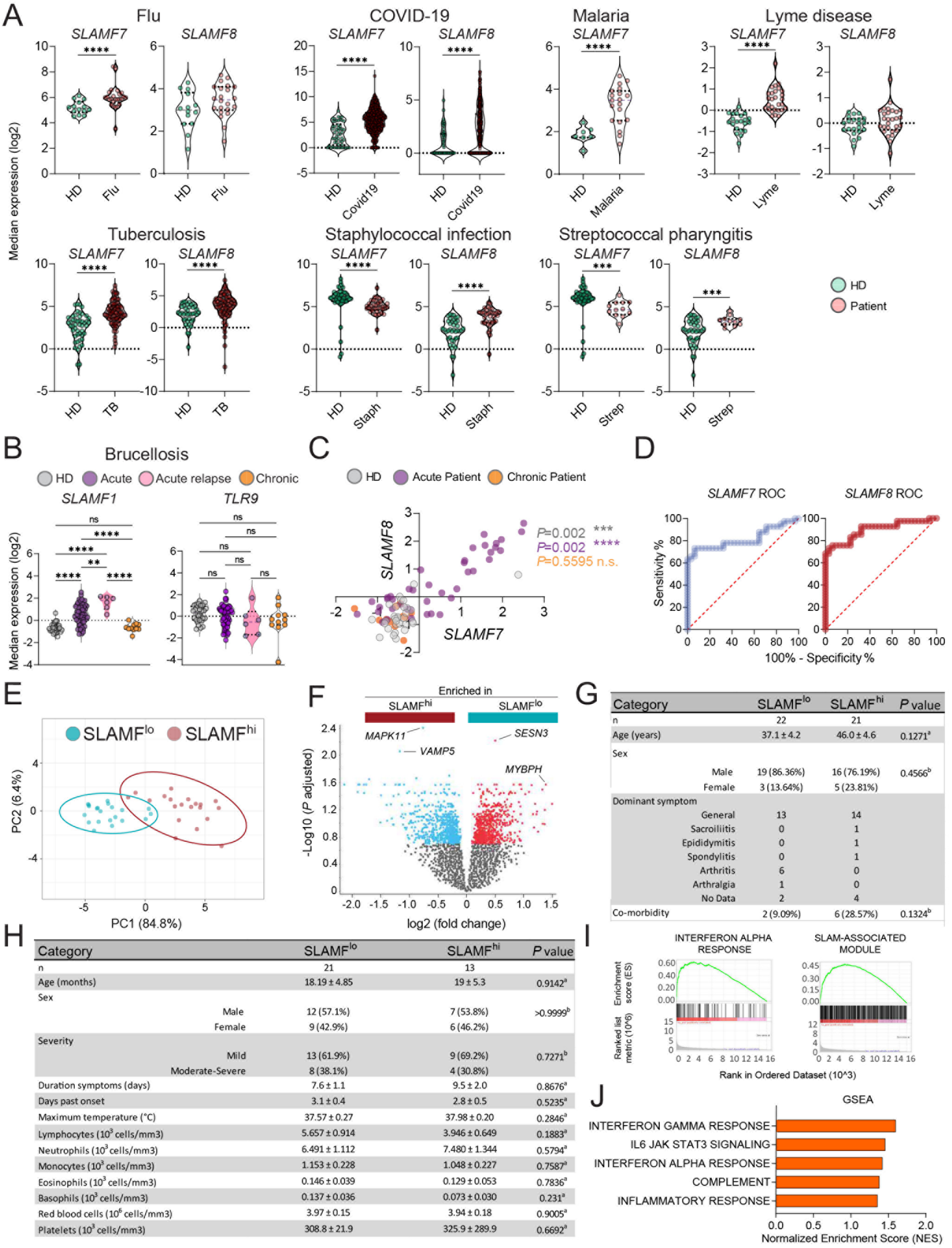

G

Category	SLAMF^{lo}	SLAMF^{hi}	*P* value
n	22	21	
Age (years)	37.1 ± 4.2	46.0 ± 4.6	0.1271[a]
Sex			
Male	19 (86.36%)	16 (76.19%)	0.4566[b]
Female	3 (13.64%)	5 (23.81%)	
Dominant symptom			
General	13	14	
Sacroiliitis	0	1	
Epididymitis	0	1	
Spondylitis	0	1	
Arthritis	6	0	
Arthralgia	1	0	
No Data	2	4	
Co-morbidity	2 (9.09%)	6 (28.57%)	0.1324[b]

H

Category	SLAMF^{lo}	SLAMF^{hi}	*P* value
n	21	13	
Age (months)	18.19 ± 4.85	19 ± 5.3	0.9142[a]
Sex			
Male	12 (57.1%)	7 (53.8%)	>0.9999[b]
Female	9 (42.9%)	6 (46.2%)	
Severity			
Mild	13 (61.9%)	9 (69.2%)	0.7271[b]
Moderate-Severe	8 (38.1%)	4 (30.8%)	
Duration symptoms (days)	7.6 ± 1.1	9.5 ± 2.0	0.8676[a]
Days past onset	3.1 ± 0.4	2.8 ± 0.5	0.5235[a]
Maximum temperature (°C)	37.57 ± 0.27	37.98 ± 0.20	0.2846[a]
Lymphocytes (10^3 cells/mm3)	5.657 ± 0.914	3.946 ± 0.649	0.1883[a]
Neutrophils (10^3 cells/mm3)	6.491 ± 1.112	7.480 ± 1.344	0.5794[a]
Monocytes (10^3 cells/mm3)	1.153 ± 0.228	1.048 ± 0.227	0.7587[a]
Eosinophils (10^3 cells/mm3)	0.146 ± 0.039	0.129 ± 0.053	0.7836[a]
Basophils (10^3 cells/mm3)	0.137 ± 0.036	0.073 ± 0.030	0.231[a]
Red blood cells (10^6 cells/mm3)	3.97 ± 0.15	3.94 ± 0.18	0.9005[a]
Platelets (10^3 cells/mm3)	308.8 ± 21.9	325.9 ± 289.9	0.6692[a]

Supplemental Figure 1 (related to Figures 1 and 2). Upregulation of SLAMF7 and 8 at the mRNA and protein levels in several diseases and correlation with brucellosis and salmonellosis. (**A**) Violin plots show the median expression of *SLAMF7* and *SLAMF8* normalized counts from blood transcriptomics data between healthy donors (HD, Green dots) and patients (Pink dots) suffering from flu (GSE100160; patients, n=25; HD, n=14), COVID-19 (GSE152075; patients, n=430;

HD, n=54), malaria (GSE116149; patients, n=19; HD, n=7), Lyme disease (GSE145974; patients, n=39; HD, n=21), tuberculosis (GSE19491; patients, n=103; HD, n=52), staphylococcal infection (GSE100165; patients, n=99; HD, n=44), and streptococcal pharyngitis (GSE158163; patients, n=11; HD, n=51). Significant differences are shown (Mann-Whitney U test). ***, $P < 0.001$; ****, $P < 0.0001$. no *P* value, non-significant. (**B**-**G**) RNA seq transcriptomic profiling obtained from whole blood samples from healthy donor (HD) controls or primary brucellosis patients in acute, acute relapse or chronic phase of infection. (**B**) The median expression of *SLAMF1* and *TLR9* normalized counts is shown. X-axis HD controls, n=36 (Grey); Brucellosis patients, Acute, n=54 (Purple), Acute with relapse, n=6 (Pink), Chronic, n=12 (Orange). Y-axis: log2 residual gene expression counts. Significant differences are shown (Multiple comparison Kruskal-Wallis test, followed by post-hoc Dunn's test). (**C**) Correlation between *SLAMF7* and *SLAMF8* RNA counts across all groups of individuals including HD, acute and chronic brucellosis patients. Nonparametric Spearman correlation test. (**D**) Receiver operating characteristic (ROC) curves of *SLAMF7* and *SLAMF8* gene expression level's ability to discriminate acute from chronic brucellosis. (**E**-**G**) Analysis of blood transcriptomic signatures in patients categorized according to their level of expression of SLAMF7 and 8, and for which SLAMF refers to SLAMF7 and 8 only. SLAMF^{hi}, n=21; SLAMF^{lo}, n=22. (**E**) PCA analysis of blood signature in SLAMF^{hi} and SLAMF^{lo} categorized brucellosis patients. (**F**) Volcano plot showing differentially expressed genes between SLAMF^{hi} and SLAMF^{lo} brucellosis patients. (Light Blue: enriched in SLAMF^{hi} patients, Red: enriched in SLAMF^{lo} patients). (**G**) Demographic and clinical data of SLAMF^{hi} and SLAMF^{lo} categorized brucellosis patients**.** Continuous data are expressed as mean ± SD, and categorical data are expressed as number (percentages). *P* values were calculated by (a) Mann-Whitney U test for unpaired and non-parametric samples and by (b) Fisher exact test for categorical variables. (**H**) Demographic and clinical data of SLAMF^{hi} and SLAMF^{lo} categorized salmonellosis patients from the second cohort (GSE69529). Continuous data are expressed as mean ± SEM, and categorical data are expressed as number (percentages). *P* values were calculated by (a) Mann-Whitney U test for unpaired and non-parametric samples and by (b) Fisher exact test for categorical variables. (**I**, **J**) Gene set enrichment analysis (GSEA) was performed on RNA-Seq data from SLAMF^{hi} and SLAMF^{lo} categorized salmonellosis patients (GSE69529). SLAMF^{hi}, n=13; SLAMF^{lo}, n=21. (**I**) Individual gene set enrichment plots for Interferon alpha response and SLAM-associated module are shown. (**J**) Top five gene sets according to the normalized enrichment score (NES). FDR q-val<0.05.

Supplemental Figure 2 (related to Figures 3 and 4)

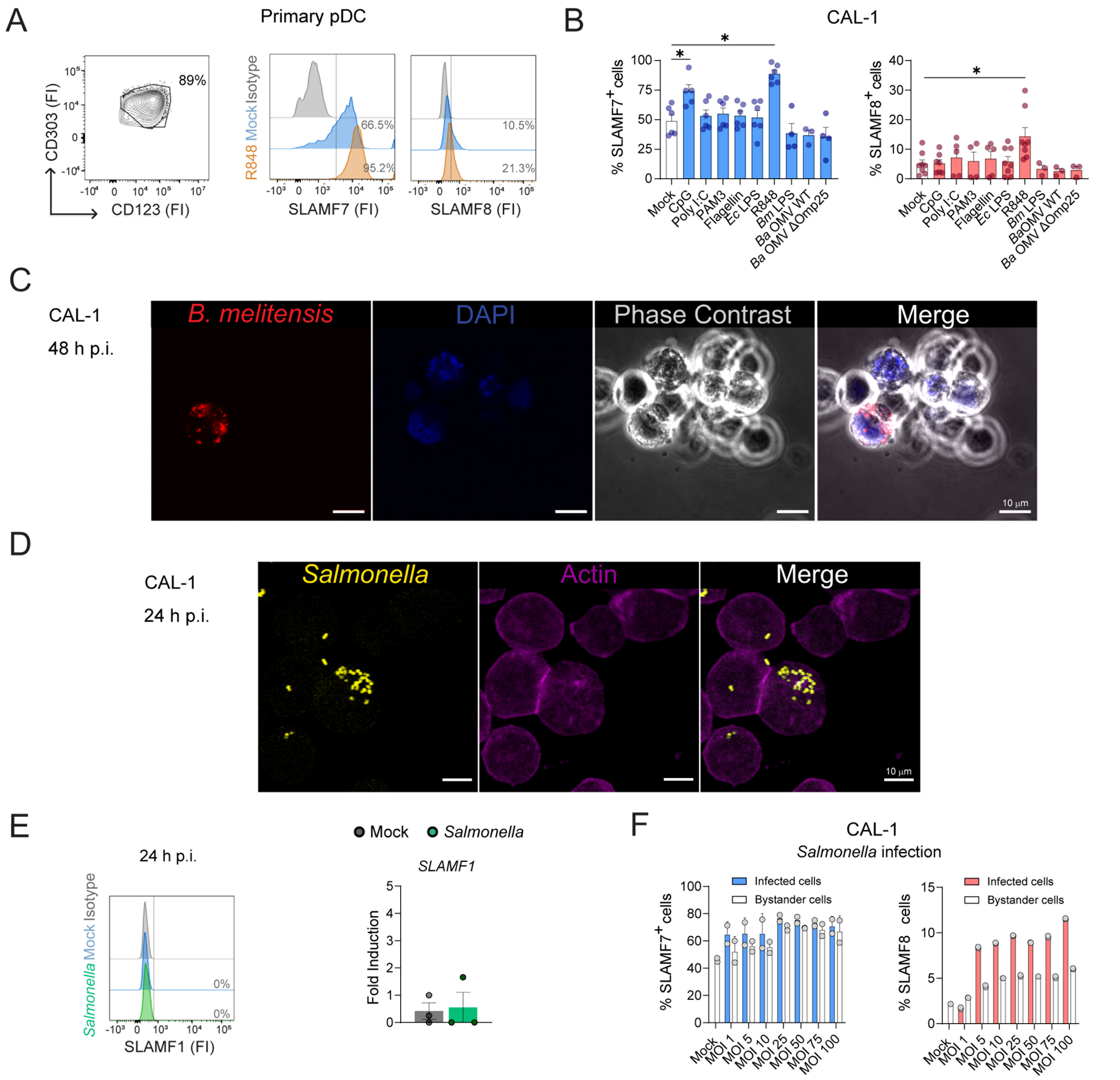

Supplemental Figure 2 (related to Figures 3 and 4). Inducibility of SLAMF7, SLAMF8 and SLAMF1 in human pDC, and infectability of CAL-1 cells. (**A**) Human plasmacytoid dendritic cells (pDC) were purified from healthy individual's blood. pDC purity was analyzed by flow cytometry based on the expression of CD303 and CD123 (left panel). Then, cells were stimulated (Orange) or not (Blue) with R848 (100 ng/mL) for 24 h, and SLAMF7 and SLAMF8 expression was evaluated by flow cytometry. Representative histograms of flow cytometry experiments showing SLAMF7 (middle) and SLAMF8 expression (right) are shown. (**B**) CAL-1 cells were stimulated with CpG (TLR9 ligand, 100 ng/mL), PolyI:C (TLR3 ligand, 100 ng/mL), PAM3CSK4 (TLR1/2 ligand, 100 ng/mL), flagellin (TLR5 ligand, 100 µg/mL), *E. coli* LPS (*Ec* LPS, TLR4 ligand, 100 ng/mL), R848 (TLR7/8 ligand, 100 ng/mL), *B. melitensis* LPS *(Bm* LPS, 10 ng/mL), WT *B. abortus* outer membrane vesicles (*Ba* OMV WT, 10 µg/mL), or ΔOmp25 *B. abortus* outer membrane vesicles (*Ba* OMV ΔOmp25, 10 µg/mL) for 24 h. Then, SLAMF7 and SLAMF8 expression was evaluated by flow cytometry. Column graphs showing the percentage of SLAMF7^{+} (left, Blue) and SLAM/8^{+} (right, Pink) cells. Mean ± SD. n=8. Significant differences are indicated. Statistical differences were all

calculated using One-way ANOVA followed by Dunnett's multiple comparisons test. *, $P < 0.05$. (**C**) Representative confocal images showing CAL-1 cells infected with mCherry-*Brucella melitensis* (M.O.I. of 5000) at 48 h post infection (p.i.). Scale bars: 10 µm. n=3. (**D**) Representative confocal images of CAL-1 infected with DsRed-*Salmonella* Typhimurium at 24 h p.i.. *Salmonella* (Yellow) and Actin (Purple) are shown. Scale bars: 10 µm. n=3. (**E**) CAL-1 cells were infected with *S.* Typhimurium (M.O.I. of 25, Green) or not for 24 h. Then, SLAMF1 expression were evaluated by flow cytometry and RT-qPCR. n=3. Left: representative histograms of flow cytometry experiments are shown. Right: Column graphs showing *SLAMF1* gene expression relative to housekeeping gene, *ACTB*. Mean ± SEM. (**F**) CAL-1 cells were infected with DsRed-*S.* Typhimurium at the indicated M.O.I. for 24 h. Then, SLAMF7 and SLAMF8 expression was evaluated by flow cytometry on infected (filled colored bars; SLAMF7$^+$, Blue; SLAMF8$^+$, Pink) and bystander cells (empty bars). Column graphs showing the percentage of SLAMF7$^+$ and SLAMF8$^+$ cells. Mean ± SD. n=3. Statistical differences were all calculated using One-way ANOVA followed by Dunnett's multiple comparisons test. no *P* value, non-significant.

Supplemental Figure 3 (related to Figure 5)

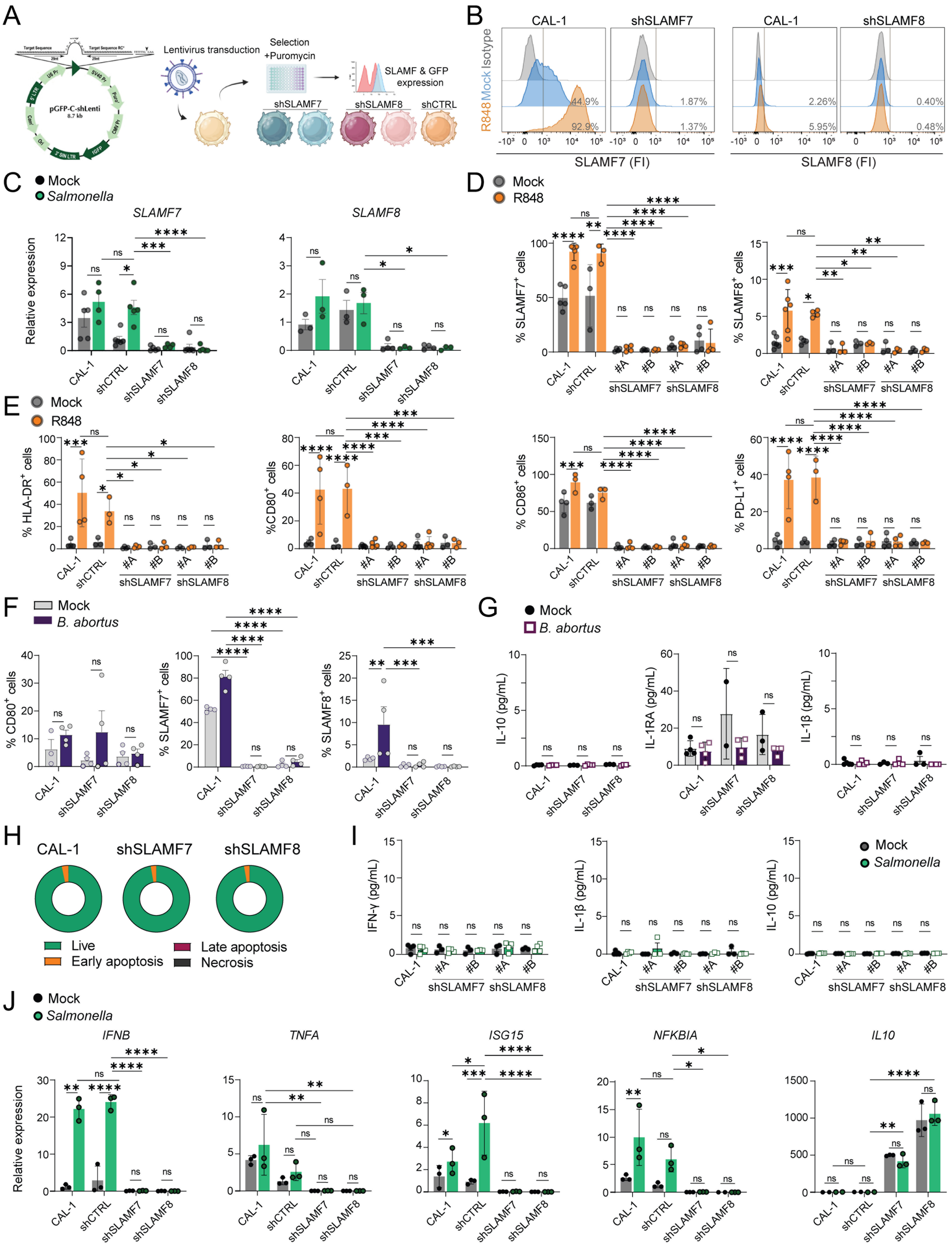

Supplemental Figure 3 (related to Figure 5). Generation and analysis of SLAMF7- or SLAMF8-silenced CAL-1 cells. (**A**) Strategy for generating and selecting stable specific SLAMF7 and SLAMF8-knockdown (KD) CAL-1 cells using lentivirus transduction. (**B**) CAL-1 cells and SLAMF-silenced cells (shSLAMF7 and shSLAMF8) were stimulated with the TLR7 ligand R848 (100 ng/mL) for 24 h. Representative histograms showing SLAMF7 and SLAMF8 surface expression demonstrate efficient

SLAMF7- or SLAMF8-silencing in CAL-1 cells at resting state (Mock, Blue) and upon stimulation (R848, Orange). (**C**) CAL-1, non-targeting shRNA-transduced control (shCTRL) and SLAMF-silenced cells were infected (Green) or not (Grey) with DsRed-*Salmonella* Typhimurium (M.O.I. of 25) for 3 h. SLAMF7 (left) and SLAMF8 (right) gene expression was evaluated by RT-qPCR. Column graphs showing relative gene expression to housekeeping gene, *ACTB*. Mean ± SD. n=3-5. (**D** and **E**) CAL-1 cells, non-targeting shRNA-transduced control cells (shCTRL) and SLAMF-silenced cells (shSLAMF7 and shSLAMF8, 2 independent clones, A and B, analyzed per type of SLAM-KD cells) were stimulated (Orange) or not (Grey) with the TLR7 ligand R848 (100 ng/mL) for 24 h. Expression of SLAMF7 (**D**, left) and SLAMF8 (**D**, right) and HLA-DR, CD80, CD86 and PD-L1 surface markers (**E**, from left to right) were determined by flow cytometry. Column graphs showing the percentage of positive cells. Mean ± SD. n=4-5. (**F** and **G**) CAL-1 and SLAMF-KD cells were infected with mCherry-*Brucella abortus* or -*B. melitensis* (M.O.I. of 5000, Purple) for 48 h. (**F**) Surface expression of CD80, SLAMF7 and SLAMF8 was determined by flow cytometry. Column graphs showing the percentage of positive cells. Mean ± SD. n=4. (**G**) Cytokine secretion (pg/ml) was determined in culture supernatants from F by multiplex assay and shown here by column graphs. Mean ± SD. n=3-4. (**H**-**J**) CAL-1 and SLAMF-silenced cells were infected with DsRed-*Salmonella* Typhimurium (M.O.I. of 25) for 24 h (**H** and **I**) or 3 h (**J**). (**H**) No change in viability was observed in wild-type or silenced CAL-1 cells. Cells were subjected to dual staining with annexin V (Ann V) and propidium iodide (PI), and cell death was assessed by flow cytometry. Parts of the whole represent the percentage of live cells (Green) or cells in early apoptosis (Ann V^+ PI^-, Orange), late apoptosis (Ann V^+ PI^+, Pink) or necrosis (Ann V^- PI^+, Purple). (**I**) No IFN-γ, IL-1β or IL-10 secretion was triggered in human pDC by *S*. Typhimurium infection. Cytokine secretion was determined in culture supernatants using multiplex assay. Column graphs showing cytokine concentration (pg/ml). Mean ± SD. n=4. (**J**) *IFNB*, *TNFA*, *ISG15*, *NFKBIA*, and *IL10* gene expression was evaluated by RT-qPCR. Column graphs showing relative gene expression to housekeeping gene, *ACTB*. Mean ± SD. n=3. Statistical differences were all calculated using Two-way ANOVA followed by Sidak's multiple comparisons test. *, $P < 0.05$; **, $P < 0.01$; ***, $P < 0.001$, ****; $P < 0.0001$. ns, non-significant.

Supplemental table 1. COVID-19 cohort features.

**Demographic characteristics**		**Patients**	**HD**
Sex, no. (%)		n = 430	n = 54
	Male	176	30
	Female	201	24
	Unknown	53	0
Age (years)	Median (range)	54 (2-98)	46.5 (12-90)
**Clinical characteristics**		**Patients**	**HD**
Viral Load, no.	Low	99	N/A
	Medium	206	N/A
	High	108	N/A
	Unknown (not analyzed)	17	N/A

Database and Identifier: GEO - GSE152075. N/A: Not applicable.

Supplemental table 2. Flu cohort features.

**Demographic characteristics**		**Patients**	**HD**
Sex, no. (%)		n = 25	n = 14
	Male	12 (48)	7 (50)
	Female	13 (52)	7 (50)
Age (years)			
	Median (IQR)	4.0 (1.6-7.5)	3.4 (1.5-6.9)
**Clinical characteristics**		**Patients**	**HD**
Co-infection	Yes	6	N/A
	No	19	N/A
Days from symptoms to recruitment: median (IQR)		5 (3.0-7.0)	N/A
Intensive care required, no. (%)		12 (48)	N/A

Database and Identifier: GEO - GSE100160. N/A: Not applicable.

Supplemental table 3. Malaria cohort features.

**Demographic characteristics**		**Patients**	**HD**
Sex, no. (%)		n = 19	n = 7
	Male	10 (52.6)	4 (57.1)
	Female	9 (47.4)	3 (42.9)
Age (years)			
	Range	29-48	Age-matched controls
**Clinical characteristics**		**Patients**	**HD**
Symptoms, no. (%)	Episodic Fever	18 (94.7)	N/A
	Chills	13 (68.4)	N/A
	Vomiting	12 (63.0)	N/A
	Headache	13 (68.4)	N/A

Database and Identifier: GEO - GSE116149. N/A: Not applicable.

Supplemental table 4: Lyme disease cohort features.

**Demographic characteristics**		**Patients**	**HD**
Sex, no. (%)		n = 39	n = 21
	Male	22 (56)	9 (43)
	Female	17 (44)	12 (57)
Age, no. (%)			
	<60 years	28 (68)	16 (76)
	≥60 years	11 (28)	3 (14)
**Clinical characteristics**		**Patients**	**HD**
EM rash	Median size, cm2 (range)	104 (11–1,440)	N/A
	Median duration, days (range)	5 (1–60)	N/A
	MEM, no. (%)	26 (67)	N/A
Blood culture for *B. burgdorferi*	Positive	29 (74)	N/A
Disseminated infection	No. (%) with MEM and/or positive blood culture	38/39 (95)	N/A

Database and Identifier: GEO - GSE145974. N/A: Not applicable.

Supplemental table 5. Tuberculosis cohort features.

**Demographic characteristics**		**Patients**	**HD**
Sex, no. (%)		n = 103	n = 52
	Male	67 (65)	22 (43)
	Female	36 (35)	30 (57)
Age (years)			
	Mean (SD)	35.2 (13.4)	31.8 (9.1)
	Median	32	29
**Clinical characteristics**		**Patients**	**HD**
Sputum smear, no	+	15	N/A
	++	6	N/A
	+++	14	N/A
	Nil	16	N/A
	n.d.	30	N/A
Modal X-ray grade	Minimal	12	N/A
	Moderate	24	N/A
	Advanced	23	N/A
	n.d.	44	N/A
Isolate sensitivity	Fully sensitive	45	N/A
	Resistant	16	N/A
	n.d.	42	N/A
BCG vaccinated	Yes	67	46
	No	25	6
	Not known	11	-

Database and Identifier: GEO - GSE19491. N/A: Not applicable.

Supplemental table 6. Staphylococcal Infection cohort features.

**Demographic characteristics**		**Patients**	**HD**
Sex, no. (%)		n = 99	n = 44
	Male	58 (59)	22 (50)
	Female	41 (41)	22 (50)
Age (years)			
	Mean (SD)	6.9 (4.9)	7.0 (4.8)
	Median	7	7
**Clinical characteristics**		**Patients**	**HD**
Pathogen, no. (%)	MSSA	19 (19)	N/A
	MRSA	80 (81)	N/A
Condition, no. (%)	Pneumonia	21 (21)	N/A
	Bacteremia	82 (83)	N/A
	Osteomyelitis	82 (83)	N/A
	Pyomyositis	21 (21)	N/A

Database and Identifier: GEO - GSE100165. N/A: Not applicable.

Supplemental table 7. Streptococcal Pharyngitis cohort features.

Demographic characteristics		Patients	HD
Sex, no. (%)		n = 11	n = 51
	Male	1 (9)	24 (47)
	Female	10 (91)	27 (53)
Age (years)			
	Mean (SD)	10.4 (5.2)	13.4 (2.7)
	Median	8.5	13.1
**Clinical characteristics**		**Patients**	**HD**
GAS culture and NAT results no. (%)	GAS throat culture	7 (64)	N/A
	NAT - Throat swab	7 (64)	N/A
	NAT - Throat wash	7 (64)	N/A
	NAT - Saliva	8 (73)	N/A

Database and Identifier: GEO - GSE158163. N/A: Not applicable.

Supplemental table 8. Brucellosis cohort features.

**Demographic characteristics**		**Patients**	**HD**
Sex, no. (%)		n = 73	n = 30
	Male	61 (84)	Sex-matched
	Female	12 (16)	controls
Age (years)			
	Mean (SD)	39.9 (17.3)	Age-matched
	Median	40	controls
**Clinical characteristics**		**Patients**	**HD**
Disease stage, no.	Acute	56	N/A
	Acute with Relapse	6	N/A
	Chronic	11	N/A
Dominant symptom, no.	General	51	N/A
	Sacroiliitis	1	N/A
	Epididymitis	2	N/A
	Spondylitis	1	N/A
	Arthritis	6	N/A
	Arthralgias	1	N/A
	No Data	11	N/A

Database and Identifier: GEO - GSE69597. Not applicable.

Supplemental table 9. Salmonellosis cohort 1 features.

**Demographic characteristics**		**Patients**	**HD**
Sex, no. (%)		n = 31	n = 47
	Male	15 (49)	22 (47)
	Female	16 (51)	25 (53)
Age (years)			
	Median (IQR)	20.0 (15.0-27.0)	Age-matched controls
**Clinical characteristics**		**Patients**	**HD**
Temperature (°C)		38.8 (38.3−39.4)	N/A
Days of illness before enrolment		5.0 (4.0−6.0)	N/A
Typhoid vaccination, no. (%)		2 (6.5)	N/A
Symptoms, no. (%)	Fever	31 (100)	N/A
	Cough	10 (32)	N/A
	Constipation	2 (6.5)	N/A
	Headache	21 (84)	N/A
	Diarrhea	7 (22.5)	N/A
Culture confirmed	*S*. Typhi	19 (61)	N/A
	S. Paratyphi	12 (39)	N/A

Database and Identifier: GEO - GSE113866. N/A: Not applicable.

Supplemental Table 10. Salmonellosis cohort 2 features.

**Demographic characteristics**		**Patients**	**HD**
Sex, no. (%)		n = 34	n = 29
	Male	19 (56)	12 (41)
	Female	15 (44)	17 (59)
Age (months)			
	Mean (SD)	18.5 (20.8)	30.5 (22.8)
	Median (IQR)	10	20
**Clinical characteristics**		**Patients**	**HD**
Temperature (°C)		37.7	36.4
Days of illness before enrolment		3.0 (1.0-8.0)	N/A
Typhoid vaccination, no. (%)		0 (0)	0 (0)
WHO score, no. (%)	Mild	22 (64.7)	N/A
	Moderate	9 (26.5)	N/A
	Severe	3 (8.8)	N/A

Database and Identifier: GEO - GSE69529. N/A: Not applicable.

SUPPLEMENTAL METHODS

Immunofluorescence microscopy

At different time-points of infection, cells were seeded on alcian blue-treated coverslips, fixed with 3.2% PFA, permeabilized with 0.05% saponin, followed by 1 h blocking with 2% BSA in PBS. Primary antibodies (rabbit anti-LAMP1, 1/1000, kind gift of Dr. Minoru Fukuda (La Jolla Cancer Research Foundation, U.S.A.); phalloidin-AF488 for F-actin detection, #A12379 Therrmofisher) were incubated for 1 h in 1xPBS, 0.1% saponin, 0.1% horse serum. After 2 washes in 1xPBS, secondary antibodies were incubated for 45 min, and coverslips were finally mounted in Prolong Gold antifade reagent with DAPI (#P36931 Invitrogen Thermofisher). Images (of 1024x1024 pixels) were acquired on a Leica SP5 laser scanning confocal microscope and assembled using Fiji software V1.53s (ImageJ).

Bacterial intracellular burden assay

To monitor bacterial intracellular load, infected cells at different time-points were washed 3 times in 1xPBS and lysed with 0.1% Triton X-100 (#X100 Sigma-Aldrich) in H_2O. Serial dilutions of cell lysates were made and 20 µl aliquots were plated in triplicates onto LB agar. Plates were incubated for 24 h at 37°C, and colonies were counted from dilutions yielding 10 to 100 visible colonies.

Cytokine measurement

For *Salmonella*- and *Brucella*-infected cells, supernatants were harvested and cytokine measured using Meso Scale Discovery (MSD) U-PLEX Biomarker Group1 (human) Assay (#K15067L-1 Meso Scale Diagnostics) for IFN-α2a, IFN-β, IFN-γ, IL-29/IFN-λ1, TNF-α, IL-10, IL-6, IL-1β, and IL-1RA. MSD assay was performed as per

manufacturer's instructions but with a 16 h incubation of the samples to improve binding to the plate. Supernatants from SLAMF7 and SLAMF8 cross-linked human primary pDC were harvested at 24h and IFN-α2a, IFN-β, IFN-γ, IL-29/IFN-λ1, IL-28A/IFN-λ2, CXCL10, TNF-α, IL-12p70, IL-1β, IL-6 and IL-8 were measured using LEGENDplex assay (#741270 Hu Anti-Virus Response Panel V02, Biolegend) according manufacturer's instructions.

For human brucellosis studies, cytokine/chemokine concentrations were determined in serum harvested at initial visit from acute, acute with relapse, chronic infected patient and HD groups by Luminex using the Human Cytokine/Chemokine/Growth Factor 45-Plex ProcartaPlex Panel 1 (#EPX450-12171-901 ThermoFisher) according supplier's recommendations. The resulting data were normalized and analyzed by OPLS-DA (Orthogonal Partial Least Squares Discriminant Analysis) using the MetaboAnanalyst platform version 5.0.

ROS detection

To measure ROS, CAL-1 cells were infected with *Salmonella* for 4 h and then incubated for 30 min at 37 °C with CellROX Deep Red Reagent (1 μM, #C10422 Invitrogen Thermofisher) for total (cytoplasmic and nuclear) cellular ROS assessment or with MitoSOX (1,25 μM, #M36009 Invitrogen Thermofisher) in PBS 2%FBS for mtROS detection. Cells were then washed twice with warm PBS, immediately re-suspended in cold PBS containing 1% FBS and subjected to flow cytometry analysis.

To analyze the oxidative stress detected by the bacteria, *Salmonella* WT- and *Salmonella* ROS sensing strain-infected CAL-1 cells were washed twice with PBS, lysed with 0.1% Triton X-100 in PBS and immediately fixed in two volumes of 3%

paraformaldehyde for 1 h. Large debris and nuclei were removed by centrifugation for 5 min at 200 g and bacteria were pelleted at 20,000 g for 10 min. Bacteria were resuspended in 40 µL of 10 mM NH_4Cl in PBS, immunolabelled with a mouse anti-*Salmonella* 1E6 (#MA1-83451 Thermofisher) and a rat anti-mouse IgG1 BV421, both diluted 1:1000 and analyzed by flow cytometry.

RNA extraction and RT-qPCR

Total RNA was extracted from infected cells using TRIzol (#15596026 Thermofisher) and Direct-zol RNA microprep Kit (#ZR2060 Zymo Research Ozyme) following the manufacturer's instructions with an additional on-column DNase RNase-free (#79254 Qiagen) incubation step to get rid of any genomic DNA trace. cDNA were generated with 500 ng of RNA as a template using QuantiTech Reverse Transcription Kit (#205311 Qiagen) according to manufacturer's recommendations. 2 µl of cDNA corresponding to 6 ng of starting total RNA were used for qPCR with specific primer sets. Amplification reactions were performed in duplicates with SYBR Green mix (#RR420L Takara) in 7500Fast Real-time PCR machine (Applied Biosystems). Gene encoding β-actin, *ACTB*, was selected as the best housekeeping gene in human pDC. Data were normalized with this gene, further analyzed by the ΔΔCt comparative cycle threshold method and presented as relative expression versus Mock-treated cells. Primer sequences were *SLAMF7* forward 5'-TGCCTCACCCTCATCTATATCCT-3' and reverse 5'-CTTCAGGGGGAAAGTCACGG-3', *SLAMF8* forward 5'-CTCCGTGTTGATGGTGGACA-3' and reverse 5'- TGAACACTTGTACCACGGGC-3', *IFNB* forward 5'-ACCATCTGAAGACAGTCCTGG-3' and reverse 5'-GTGACTGTACTCCTTGGCCTT-3', *TNFA* forward 5'-

AGTGACAAGCCTGTAGCCCATGTT-3' and reverse 5'-GTTATCTCTCAGCTCCACGCCATT-3', *ISG15* forward 5'-GCAGCGAACTCATCTTTGCC-3' and reverse 5'-TCTTCACCGTCAGGTCCCA-3', *NFKBIA* forward 5'-CACCGAGGACGGGGACT-3' and reverse 5'-CACCTGGCGGATCACTTCC-3', *IL10* forward 5'-CTGGGGGAGAACCTGAAGAC-3' and reverse 5'-GGCCTTGCTCTTGTTTTCACA-3', *ACTB* forward 5'-CATGAGAAGTATGACAACAGCC-3' and reverse 5'-AGTCCTTCCACGATACCAAAGT-3'.

For prokaryotic RNA extraction, $5x10^9$ CFU of *S.* Typhimurium or *E. coli* were resuspended in 1 ml of TRIzol and their RNA was purified using Quick-RNA DirectZol columns ((#ZR2072 Zymo Research Ozyme) according to the manufacturer's instructions. The quantification and purity of the prokaryotic RNA was determined using a Nanodrop spectrophotometer.
